# Stereoselective synthesis and X-ray structure determination of novel 1,2-dihydroquinolinehydrazonopropanoate derivatives

**DOI:** 10.1016/j.heliyon.2024.e25248

**Published:** 2024-02-01

**Authors:** Hendawy N. Tawfeek, Ahmed M. Tawfeek, Stefan Bräse, Martin Nieger, Essmat M. El-Sheref

**Affiliations:** a Chemistry Department, Faculty of Science, Minia University, El-Minia, 61519, Egypt; b Chemistry Department, College of Science, King Saud University, Riyadh, 11451, Saudi Arabia; c Institute of Biological and Chemical Systems, IBCS-FMS, Karlsruhe Institute of Technology, 76131, Karlsruhe, Germany; d Department of Chemistry, University of Helsinki, PO Box 55, A. I. Virtasen Aukio 1, 00014, Helsinki, Finland

**Keywords:** Aza-michael addition, Hydrazono-hydrazides, Stereoselective synthesis, 4-Hydrazinyl-quinolin-2(1*H*)-Ones, Ethyl propiolate, 1,2-Dihydroquinolin-4-yl(hydrazono)propanoate and X-ray crystallography

## Abstract

A novel series of 1,2-dihydroquinolinhydrazonopropanoate have been synthesized *via* a convenient aza-Michael addition reaction between hydrazinylquinolinones and ethyl propiolate in ethanol under refluxing temperature. The structures for all obtained products were confirmed with FTIR, NMR spectrums, as well as mass spectrometry. In addition, the monoclinic structure for compounds **8a**, **8c,** and **8d** was also confirmed *via* X-ray crystallography analyses. The *E*-configuration for the obtained products was confirmed form the X-ray analysis. On the other hand, the crystal packing shows that the intermolecular and hydrogen bonds between atoms are parallel to the bc plan.

## 1 Introduction

**Fig. 1:**
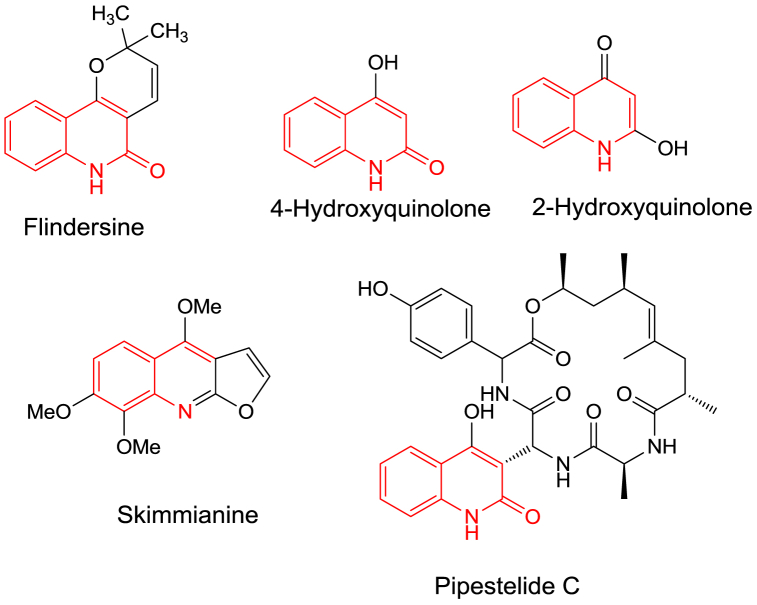
Naturally occurring quinolone-related structures.

As privileged structural subunits, quinolones have been witnessed in many biologically active compounds and natural products [[1], [2], [3]]. Moreover, 4-hydroxyquinolone, as well as *Skimmianine*, proven as an effective anti-cancer agent [4], and *Flindersine*, playing an antibacterial and antifungal activity [5], were examples of naturally extracting quinolone derivatives (Fig. 1). Quinoline derivatives possess peptide bonds like Pipestelide C that was isolated from a marine fungus **(**Fig. 1**)** [6].

**Fig. 2:**
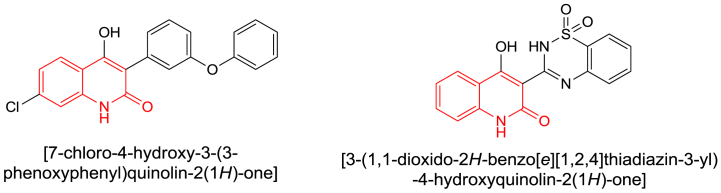
Vital drugs, including quinolinone ring.

On the other hand, there were synthetic drugs based on the main quinolinone unit on their structures, such as L-701,324, which represented a selective antagonist at the glycine site of the NMDA receptor and counteracts haloperidol-induced muscle rigidity in rats [7], the other was benzothiadiazinyl quinolinedione described to be an inhibitor of the RNA-dependent RNA polymerase enzyme transcribed by the Hepatitis C virus (Fig. 2) [8].

Douche et al. have reported the synthesis of imidazolylquinoline derivatives, which have been investigated as potential antiviral SARS-CoV-2 candidate [9].

The hydrazide-hydrazone moiety –NHN=CH has been an important structure group for various biological activities, including antibacterial, antifungal, analgesic, anti-inflammatory, antidepressant, and anti-cancer activities [[10], [11], [12]]. Using the hydrazide-hydrazone moiety in conjunction with a quinoline system, supporting their activities as antimicrobial [13], antimycobacterial [14], anti-tubercular [15,16], anticonvulsant [17], and cytotoxic activity [18,19]. These biological activities are attributed to the numerous interesting and important properties of the hydrazide-hydrazone moiety, for example, their relatively higher metabolic stability towards proteases than amides and their tunable, labile nature in acidic pH [20].

Thus, the developing efficient strategies for constructing such frameworks has attracted the growing interest of synthetic chemists. The last decade has shown significant progress in quinolone derivatives synthesis and biological evaluation, owing to their wide application in medicinal chemistry.

Elbastawesy et al. have reported the synthesis of 6-substituted quinoline-2-one thiosemicarbazides, and their activities were evaluated *invitro* against the urease-producing *R. mucilaginosa* and *Proteus mirabilis* bacteria as fungal and bacterial [21].

Valencia et al. have reported the synthesis of quinolone-based thiosemicarbazones and investigated their *invitro* activities against *Mycobacterium tuberculosis* (*M. tuberculosis*) [22]. However, the reaction of hydrazinyl quinolone with tetracyanoethylene (TCNE) was carried out by Elbastawesy et al. to afford pyrazolyl quinolone derivatives and their biological activities as potential apoptotic antiproliferative agents targeting the EGFR inhibitory pathway have been investigated [23].

Recently, Al-Wahaibi et al. have constructed azaspiroquinolone derivatives *via* a one-pot reaction between hydrazinylquinolones, cyclic ketones, and thioglycolic acid, alongside studying the biological properties of the synthesized compounds as antiproliferative against four cancer cells [24].

Here, we have reported the reaction of hydrazonylquinolones as donating molecules with a π-deficient molecule ethyl propynolate, which possess a C≡C bond attached to an electron-withdrawing group. Our expected goal is to synthesize a pyrazoloquinolinone ring *via* nucleophilic attack of the hydrazinyl moiety into the triple bond followed by cyclization. Unfortunately, the analyses and the X-ray measurements clarified that 1,2-dihydroquinolinehydrazonopropanoate derivatives were formed without internal cyclization occurring. The reaction proceedes *via* nucleophilic addition of a nucleophile (QNHNH_2,_ donor) on the alkyne (HC≡CCOOCH_2_CH_3,_ Michael acceptor) in agree with aza-Michael addition reaction [25,26].

## 2 Experiments

Melting points were measured by a Stuart melting point apparatus and were uncorrected. The IR spectra were recorded using an FTIR Alpha 24 spectrophotometer as KBr pellets. The ^1^H and ^13^C NMR spectra were recorded in DMSO–*d*6 as a solvent on Varian Gemini NMR spectrometer at 400 and 100 MHz, respectively, using TMS as an internal standard. Chemical shifts were reported as *δ* values (ppm), while couplings constants (*J*) are measured in hertz (Hz). Some NMR spectra were measured in DMSO-*d*_*6*_ on a Bruker spectrometer (300 MHz for ^1^H and 75 MHz for ^13^C) at MICROANALYICAL CENTER, Cairo University. Mass spectra were recorded by a MAT 95 +FAB mass spectrometer in EI (70 eV) model. The reactions were closely monitored with TLC (thin-layer chromatography) on Merck alumina-backed TLC plates Pf_254_ using UV light.

### 2.1 Chemistry

The starting materials were prepared according to the reported articles [[27], [28], [29]], with slight modification in the preparation of hydrazinylquinolones **5a-g** 4-chloroquinolones **4a-g** and were refluxed with hydrazine hydrate for 3 h. Hydrzine hydrate acted as reagent and solvent. The formed precipitate was filtered, flushed several times with water, and used for the second step without further purifications.

### 2.2 Synthesis of quinolone hydrazonopropanoates

In a round bottomed flask (50 ml), substituted hydrazinyl quinolone-2-one (1 mmol) suspended in ethanol (10 ml) ethyl propiolate (1.2 mmol) was added, the mixture was refluxed for about 6 h. The reaction was monitored with TLC, until the starting spot disappeared. The formed precipitate was filtered off, flushed several times with hot ethanol, dried, and recrystallized using methanol furnished 1,2-dihydroquinolin ehydrazono propanoate derivatives **8a-g**. Single crystals, suitable for X-ray crystallographic analyses for compounds **8b**, **8c,** and **8d**, have been obtained using methanol as the choice solvent of crystallization.

### 2.3 (E)-Ethyl 3-(2-(1-methyl-2-oxo-1,2-dihydroquinolin-4-yl)hydrazono)propanoate (8a)

Pale yellow crystals (methanol), (258 mg, 90 %), Mp 190 °C; IR (KBr): ν 3237 (NH), 3085 (aryl-CH), 2991 (str., ali-CH), 1736 (ester-CO) 1628 (amide-CO), 1602 (C=N), 1554 (C=C) cm^−1^. ^1^H NMR (300 MHz, DMSO-*d*_*6*_): *δ*_H_ = 1.22 (t, *J* = 6.9 Hz; 3H, CH_3_); 3.44 (s, 3H, N–CH_3_); 3.51–3.53 (d, *J* = 11.4 Hz; 2H, –CH_2_-CH=N) [25,30]; 4.12–4.14 (q, *J* = 6.9 Hz; 2H, CH_2_O); 6.17 (s, 1H, H-3); 7.22–7.28 (m, 1H, quinolinone-H); 7.43–7.48 (m, 1H, quinolinone-H); 7.60–7.64 (m, 1H, quinolinone-H); 7.73–78 (m, 1H, quinolinone-H); 8.06 (t, *J* = 11.4 Hz; 1H, CH=N); 10.55 (brs, 1H, hydrazono-NH) ppm. ^13^C NMR (75 MHz, DMSO-*d*_*6*_): *δ*_C_ = 14.14 (CH_3_-ethyl); 28.50 (N–CH_3_); 37.88 (*C*H_2_–CH=N); 60.49 (-CH_2_O); 93.63 (C-3); 112.82 (C-4a); 115.16 (C-8); 120.96 (C-6); 122.35 (C-5); 131.09 (C-7); 140.05 (C-8a); 141.35 (C-4); 147.44 (CH=N-); 162.03 (C-2); 169.83 (ester-CO) ppm. MS (EI, 70 eV): *m*/*z* (%) 288 [M^+^, 100].

### 2.4 (E)-Ethyl 3-(2-(2-oxo-1,2-dihydroquinolin-4-yl)hydrazono)propanoate (8b)

Pale yellow crystals (methanol), (278 mg, 92 %), Mp 245 °C; ^1^H NMR (400 MHz, DMSO-*d*_*6*_): *δ*_H_ = 1.26 (t, *J* = 8.0 Hz, 3H, CH_3_); 3.48–3.51 (d, *J* = 16.0 Hz, 2H, –CH_2_-CH=N) [30]; 4.16 (q, *J* = 8.0 Hz; 2H, CH_2_O); 6.08 (s, 1H, quinolinone-H-3); 7.10–7.18 (m, 1H, quinolinone-H); 7.26–7.31 (m, 1H, quinolinone-H); 7.45–7.55 (m, 1H, quinolinone-H); 7.78 (s, 1H, quinolinone-H); 8.08 (t, *J* = 16.0 Hz, 1H, CH=N); 10.50 (brs, 1H, hydrazono-NH); 11.00 (brs, 1H, q quinolinone-NH) ppm. ^13^C NMR (100 MHz, DMSO-*d*_*6*_): *δ*_C_ = 14.05 (CH_3_-ethyl); 37.76 (*C*H_2_–CH=N); 60.49 (-CH_2_O); 93.49 (C-3); 111.71 (C-4a); 115.93 (C-8); 120.61 (C-6); 121.87 (C-5); 130.35 (C-7); 139.32 (C-8a); 141.13 (C-4); 148.45 (CH=N-); 162.79 (C-2); 169.72 (ester-CO) ppm.

### 2.5 (E)-Ethyl 3-(2-(6-methoxy-2-oxo-1,2-dihydroquinolin-4-yl)hydrazono)propanoate (8c)

Pale yellow crystals (methanol), (278 mg, 92 %), Mp 245 °C; IR (KBr): ν 3163 (NH), 3055 (aryl-CH), 2996 (str., ali-CH), 1727 (ester-CO) 1638 (amide-CO), 1604 (C=N), 1546 (C=C) cm^−1^. ^1^H NMR (400 MHz, DMSO-*d*_*6*_): *δ*_H_ = 1.26 (t, J = 8.0, 3H, CH_3_); 3.44 (d, J = 16.0, 2H, –CH_2_-CH = ); 3.80 (s, 3H, CH_3_O); 4.14 (q, *J* = 8.0 Hz; 2H, CH_2_O); 6.06 (s, H-3); 7.20 (m, 2H, quinolinone-H); 7.52 (s, 1H, quinolinone-H); 7.74 (t, *J* = 16.0 Hz, 1H, CH=N) [30]; 10.45 (brs, 1H, hydrazono-NH); 10.95 (brs, 1H, quinolinone-NH) ppm. ^13^C NMR (100 MHz, DMSO-*d*_*6*_): *δ*_C_ = 14.05 (CH_3_-ethyl); 37.74 (*C*H_2_–CH=N); 55.59 (CH_3_O); 60.51 (-CH_2_O); 93.05 (C-3); 112.01 (C-4a); 117.74 (C-8); 119.54 (C-5); 133.55 (C-7); 140.93 (C-8a); 143.31 (C-6); 148.9 (CH=N); 153.63 (C-4); 162.39 (C-2); 169.76 (ester-CO) ppm. MS (EI, 70 eV): *m/z* (%) 303 [M^+^, 100].

### 2.6 (E)-Ethyl 3-(2-(6-methyl-2-oxo-1,2-dihydroquinolin-4-yl)hydrazono)propanoate (8d)

Pale yellow crystals (methanol), (278 mg, 92 %), Mp 245 °C; ^1^H NMR (400 MHz, DMSO-*d*_*6*_): *δ*_H_ = 1.28 (t, *J* = 8.0 Hz, 3H, CH_3_); 3.44 (d, *J* = 12.0 Hz, 2H, –CH_2_-CH=N); 4.09–4.18 (q, *J* = 8.0 Hz; 2H, CH_2_O); 6.07 (s, 1H, quinolinone-H-3); 7.13–7.20 (m, 1H, quinolinone-H); 7.28–7.33 (m, 1H, quinolinone-H); 7.73–7.82 (m, 2H, quinolinone-H and CH=N); 10.51 (brs, 1H, hydrazono-NH); 10.98 (brs, 1H, quinolinone-NH) ppm. ^13^C NMR (100 MHz, DMSO-*d*_*6*_): *δ*_C_ = 14.03 (CH_3_-ethyl); 20.58 (CH_3_); 37.76 (*C*H_2_–CH=N); 60.66 (CH_2_O); 93.60 (C-3); 111.61 (C-4a); 115.70 (C-8); 127.32 (C-5); 129.66 (C-7); 137.26 (C-6); 140.99 (C-8a); 148.32 (CH=N); 162.78 (C2); 169.12 (ester-CO) ppm. *Anal. Calcd for* C_15_H_17_N_3_O_3_: C, 62.71; H, 5.96; N, 14.63. Found: C, 62.66; H, 5.88; N, 14.52.

### 2.7 Ethyl (E)-3-(2-(6-chloro-2-oxo-1,2-dihydroquinolin-4-yl)hydrazineylidene)propanoate (8e)

Pale yellow crystals (methanol), (267 mg, 87 %), Mp 181 °C; IR (KBr): ν 3232, 3152 (NH's), 3070 (aryl-CH), 2985 (str., ali-CH), 1734 (ester-CO) 1641 (amide-CO), 1595 (C=N), 1544 (C=C) cm^−1^. ^1^H NMR (300 MHz, DMSO-*d*_*6*_): *δ*_H_ = 1.23 (t, *J* = 7.2 Hz; 3H, CH_3_); 3.42–3.3.44 (d, *J* = 11.4 Hz; 2H, –CH_2_-CH=N); 4.15 (q, *J* = 7.2 Hz; 2H, CH_2_O); 6.06 (s, 1H, H-3); 7.26–7.30 (m, 1H, quinolinone-H); 7.48–7.51 (m, 1H, quinolinone-H); 7.71–7.75 (t, *J* = 11.4 Hz; 1H, CH=N); 10.56 (brs, 1H, hydrazono-NH); 11.20 (brs, 1H, quinolinone-NH) ppm. ^13^C NMR (75 MHz, DMSO-*d*_*6*_): *δ*_C_ *δ*_C_ = 14.10 (CH_3_-ethyl); 37.83 (*C*H_2_–CH=N); 60.61 (CH_2_O); 94.33 (C-3); 112.92 (C-4a); 117.58 (C-8); 121.46 (C-5); 125.15 (C-7); 130.37 (C-6); 138.14 (C-8a); 141.78 (C-4); 147.79 (CH=N); 162.80 (C2); 169.72 (ester-CO) ppm. MS (EI, 70 eV): *m/z* (%) 307 [M^+^, 100].

### 2.8 Ethyl (E)-3-(2-(7-methyl-2-oxo-1,2-dihydroquinolin-4-yl)hydrazineylidene)propanoate (8f)

Pale yellow crystals (methanol), (245 mg, 85 %), Mp 207 °C; IR (KBr): ν 3243, 3159 (NH's), 3067 (aryl-CH), 2936 (str., ali-CH), 1733 (ester-CO) 1630 (amide-CO), 1598 (C=N), 1526 (C=C) cm^−1^. ^1^H NMR (300 MHz, DMSO-*d*_*6*_): *δ*_H_ = 1.26 (t, *J* = 7.2 Hz; 3H, CH_3_-ethyl); 2.82 (s, 3H, CH_3_); 3.42 (d, *J* = 11.4 Hz, 2H, –CH_2_-CH=N); 4.16 (q, *J* = 7.2 Hz; 2H, CH_2_O); 6.18 (s, 1H, H-3); 698 (m, 1H, quinolinone-H); 7.31 (m, 1H, quinolinone-H); 7.63 (t, *J* = 11.4 Hz; 1H, CH=N); 7.84–7.87 (m, 2H, quinolinone-H); 10.48 (brs, 1H, hydrazono-NH); 11.18 (brs, 1H, quinolinone-NH) ppm. ^13^C NMR (75 MHz, DMSO-*d*_*6*_): *δ*_C_ = 14.12 (CH_3_-ethyl); 21.18 (CH_3_); 37.86 (*C*H_2_–CH=N); 60.81 (CH_2_O); 92.88 (C-3); 112.19 (C-4a); 115.61 (C-8); 125.11 (C-6); 129.78 (C-5); 134.65 (C-7); 138.51 (C-8a); 141.83 (C-4); 148.62 (CH=N); 162.30 (C2); 168.72 (ester-CO) ppm. MS (EI, 70 eV): *m/z* (%) 287 [M^+^, 100].

### 2.9 Ethyl (E)-3-(2-(8-methyl-2-oxo-1,2-dihydroquinolin-4-yl)hydrazineylidene)propanoate (8g)

Pale yellow crystals (methanol), (251 mg, 82 %), Mp 224 °C; IR (KBr): ν 3236, 3163 (NH's), 3076 (aryl-CH), 2996 (str., ali-CH), 1737 (ester-CO) 1625 (amide-CO), 1606 (C=N), 1546 (C=C) cm^−1^. ^1^H NMR (300 MHz, DMSO-*d*_*6*_): 1.24 (t, *J* = 7.2 Hz; 3H, CH_3_-ethyl); 3.44 (d, *J* = 12.0 Hz; 2H, –CH_2_-CH=N) [30]; 2.39 (s, 3H, CH_3_); 4.16 (q, *J* = 7.2 Hz; 2H, CH_2_O); 6.06 (s, 1H, H-3); 7.09 (m, 1H, quinolinone-H); 7.35 (m, 1H, Ar–H); 7.76 (t, 12.0 Hz; 1H, CH=N); 7.85 (m, 2H, quinolinone-H); 10.08 (brs, 1H, hydrazono-NH); 10.49 (brs, 1H, quinolinone-NH) ppm. ^13^C NMR (75 MHz, DMSO-*d*_*6*_): *δ*_C_ = 14.09 (CH_3_-ethyl); 17.63 (CH_3_); 37.83 (*C*H_2_–CH=N); 60.55 (CH_2_O); 93.51 (C-3); 111.64 (C-4a); 119.77 (C-5); 120.47 (C-6); 123.80 (C-8); 131.69 (C-7); 137.64 (C-8a); 141.27 (C-4); 148.95 (CH=N); 163.09 (C2); 169.77 (ester-CO) ppm. MS (EI, 70 eV): *m/z* (%) 307 (M^+^+H_2_O, 35); 289 (M^+^, 15), 190 (3).

### 2.10 Single crystal X-ray structure determination of 8b, 8c, and 8d

Single crystals generated by slow evaporation from methanol. A good crystal with a suitable size was selected for X-ray diffraction analysis.

The single-crystal X-ray diffraction study was carried out on a Bruker D8 Venture diffractometer with a PhotonII detector at 173(2) K or 298(2) K using Cu-Kα radiation (*λ* = 1.54178 Å). Dual space methods (SHELXT) [31] were employed for the structure solution, and refinement was carried out using SHELXL-2014 (full-matrix least-squares on *F*^*2*^) [32]. Hydrogen atoms were refined using a riding model (H(O) free, except MeOH in **8b**). Semi-empirical absorption corrections were applied; for **8b,** an extinction correction was applied.

### 2.11 Compound **8b**

C_14_H_15_N_3_O_3_·0.5(CH_4_O)·0.5(H_2_O), Mr = 298.32 g mol^−1^, blocks yellow, size = 0.12 × 0.06 × 0.04 mm, monoclinic, *C2*/*c* (no.15), *a* = 16.981 (6) Å, *b* = 11.048 (4) Å, *c* = 16.479 (5) Å, *β = 98.60 (2)°*, λ = 1.54178 Å, V = 3056.8 (18) Å^3^, Z = 8, D_calcd_ = 1.296 Mg m^−3^, F(000) = 1264, μ = 0.80 mm^−1^, T = 298 K, 13838 measured reflections (2θ_max_ = 144.2^o°^) 2991 independent reflections [R_int_ = 0.049], 210 parameters and 148 restraints, R_1_ [for 2495 reflections with *I* > 2σ(*I*)] = 0.056 w*R*_2_ (for all data) = 0.177, S = 1.05, largest diff. peak and hole = 0.27 e Å^−3^/-0.23 e Å^−3^.

### 2.12 Compound **8c**

C_15_H_17_N_3_O_4_, Mr = 303.32 g mol^−1^, plates yellow, size = 0.16 × 0.12 × 0.02 mm, monoclinic, space group *P2*_*1*_*/*n (no.14), *a* = 14.6490 (9) Å, *b* = 7.0414 (5) Å, *c* = 15.5731 (10) Å, *β* = 115.737 (2)°, λ = 1.54178, Å V = 1447.00 (17) Å^3^, Z = 4, D_calcd_ = 1.392 Mg m^−3^, F(000) = 640, μ = 0.86 mm^−1^, T = 173 K, 14391 measured reflections (2θ_max_ = 144.4°), 2838 independent reflections [R_int_ = 0.056], 206 parameters, 2 restraints, R_1_ [for 2549 reflections with *I* > 2σ(*I*)] = 0.050, w*R*_2_ (for all data) = 0.144, S = 1.04, largest diff. peak and hole = 0.32 e Å^−3^/-0.32 e Å^−3^.

### 2.13 Compound **8d**

C_15_H_17_N_3_O 1.5(H_2_O), Mr = 314.34, g mol^−1^, plates yellow, size = 0.35 0.25 × 0.20 mm, monoclinic, space group I (no.15), *a* = 17.8791 (3) Å, *b* = 11.1773 (2) Å, *c* = 16.4921 (2) Å, *β* = 100.178 (1)°, λ = 1.54178, Å V = 3243.92 (9) Å^3^, Z = 8, D_calcd_ = 1.287 Mg m^−3^, F(000) = 1336, μ = 0.80 mm^−1^, T = 298 K, 16724 measured reflections (2θ_max_ = 144.4°), 3185 independent reflections [R_int_ = 0.056], 214 parameters, 169 restraints, R_1_ [for 2799 reflections with *I* > 2σ(*I*)] = 0.069, w*R*_2_ (for all data) = 0.215, S = 1.08, largest diff. peak and hole = 0.42 e Å^−3^/-0.44 e Å^−3^.

CCDC 2256765 (**8b**), 2256766 (**8c**), and 2256767 (**8d**) contain supplementary crystallographic data for this paper. These data can be obtained free of charge from The Cambridge Crystallographic Data Center *via* www.ccdc.cam.ac.uk/data_request/cif (deposited at the CSD April 17, 2023).

## 3 Results and discussion

**Scheme 1:**
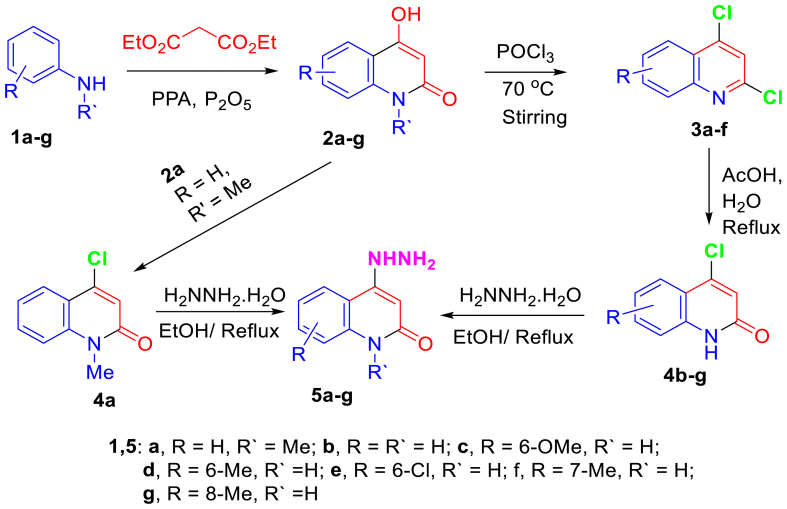
Strategy for the synthesis of hydrazonoquinolone **5a-g**.

**Scheme 2:**
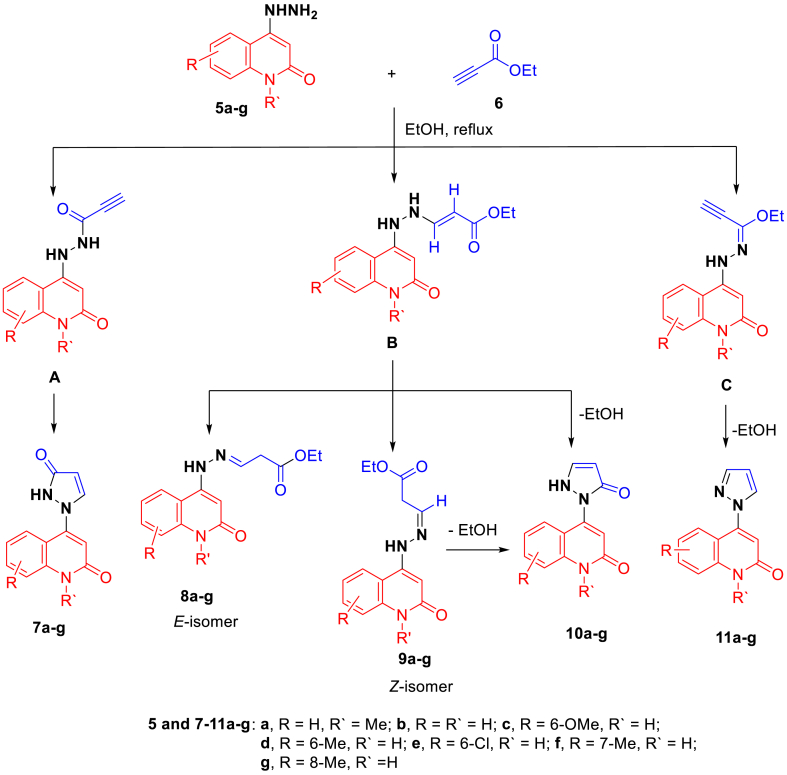
Stereoselective interaction of hydrazinylquiolinone **5a-g** with ethyl propiolate **6** and synthesis of 1,2-dihydroquinolinehydrazonopropanoate derivatives **8a-g**.

To approach the synthesis of the targeted molecules, multi-synthetic steps had been carried out [[27], [28], [29]], as shown in Scheme 1. The reactions between 4-hydrazinylquinolin-2(1*H*)-ones **5a-g** and ethyl propiolate (**6**) were carried out in ethanol under refluxing conditions to affored the corresponding 3-(2-(2-oxo-1,2-dihydroquinolin-4-yl)hydraz-ono)propanoate **8a-g** (Scheme 2). The reactions were prolonged under basic conditions to promote the cyclization to the pyrazole ring. Unfortunately, the reaction was proceed to without cycliziation and give the only open-chain products **8a-g** as a sole product.

**Fig. 3:**
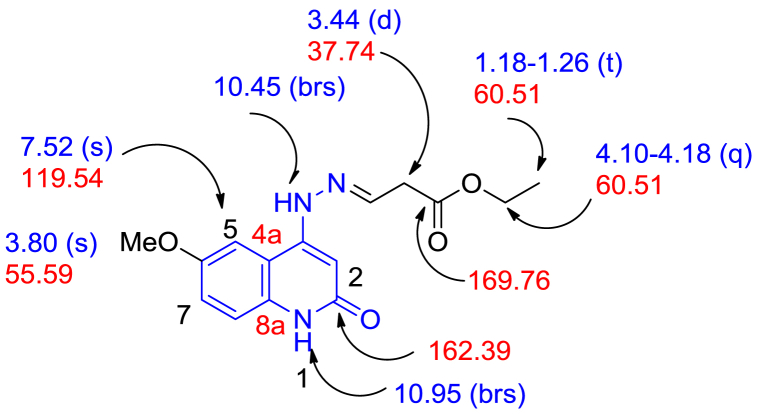
Chemical structure for (*E*)- ethyl 3-(2-(6-methoxy-2-oxo-1,2-dihydroquinolin-4-yl)hydrazinylidene)propanoate (**8c**).

The chemical composition of all the prepared compounds obtained are proved using modern spectroscopic methods such as: FTIR, ^1^H NMR, ^13^C NMR spectrum, mass spectrometry, elemental analysis, and as well as X-ray crystallography. Compound **8c** is selected as an example, which is assigned as (*E*)-ethyl 3-(2-(6-methoxy-2-oxo-1,2-dihydroquinolin-4-yl)hydrazono)propanoate (Fig. 3). The IR spectra of compound **8c**; have shown that the NH-group were observed at ν 3256 cm^−1^. Moreover, an aromatic-CH stretching vibrated at ν 3085 cm^−1^, and the aliphatic-CH at ν 2996 cm^−1^. Also, other peaks appeared at ν 1727, 1638, and 1604 cm^−1^, which assigned for (C=O ester), (C=O, quinolinone-C2), and C=N, respectively. The ^1^H NMR spectra of compound **8c** showed four singlet signals at *δ*_H_ 3.80, 6.06, 7.52, 10.45, and 10.95 ppm, which were assigned as methoxy group, quinolinone-H-3, quinolone-H-5, hydrazone-NH and quinolinone-NH, respectively. The other characteristic signals for the ethyl group at *δ*_H_ 1.26 (t, *J* = 8.0 Hz, 3H, CH_3_) and 4.14 ppm (q, *J* = 8.0 Hz, 2H, CH_2_O). The presence of the ethyl group was confirmed from the ^13^C NMR, which gives characteristic signals at *δ*_C_ 14.05 and 60.51 ppm for CH_3_ and CH_2_O- groups, respectively. Furthermore, two different carbonyl groups are resonated at *δ*_C_ 162.39 and 169.76 ppm, respectively, as quinolinone-C-2 and ester-CO. On the other hand, the mass spectrometry and elemental analysis confirmed that compound **8c** has a molecular formula C_15_H_17_N_3_O_4_ with *m*/*z* = 303, which confirmed that compound **8c** comes *via* interaction between 1 mol of 4-hydrazinyl-6-methoxyquinolin-2(1*H*)-one (**5c**) with 1 mol of ethyl propiolate (**6**) without any elimination as shown in Scheme 2.

Regarding the composition of ethyl propiolate (**6**), we notice that it contains two active centers, namely the carbonyl group (C=O) and the triple bond (C≡C); therefore, it is possible to form analogues of the compound during the intermediates **A**-**C**. If the reaction proceeded *via* intermediate **A**, the pyrazolylquinolin-2(1*H*)-ones **7a-g** would form; again, if the reaction proceeded through the intermediate **B**, our products **8a-g** and Z-form **9a-g** and pyrazolylquinolin-2(1*H*)-ones **10a-g**, would be obtained, but if the reaction proceeded through the intermediate **C**, the reaction would give the corresponding pyrazolylquinolin-2(1*H*)-ones **11a-g**. The possibility of forming these compounds is referred to as the stereoselectivity phenomena; the compounds **7a-g**, **10a-g,** and **11a-g** were ruled out at first sight according to ^1^H NMR and ^13^C NMR spectrum, in addition to the mass spectrometry. In addition, to distinguish between the two isomeric structures **8a-g** and **9a-g**, which comes from the reaction between 4-hydrazinylquinolin-2(1*H*)-ones **5a-c** and mole of ethyl propiolate (**6**) through the intermediate **B** as shown in Scheme 2.

**Fig. 4:**
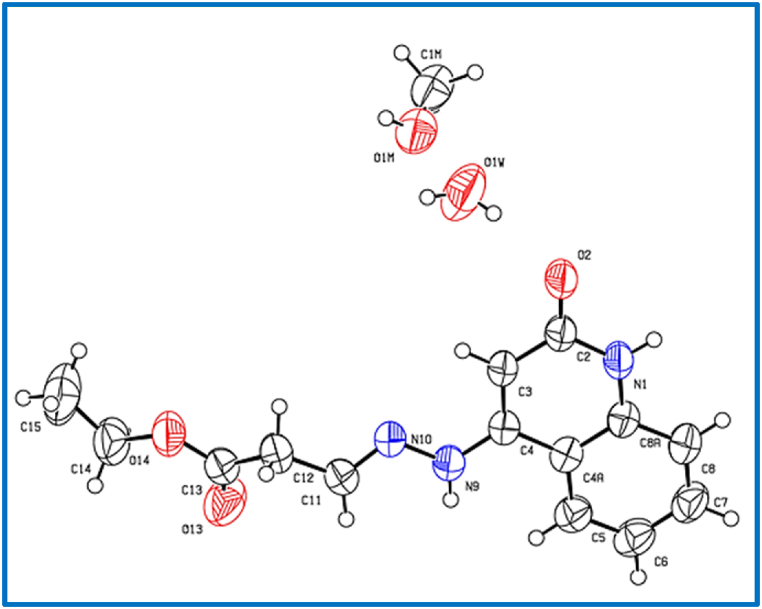
X-Ray crystallographic molecular structure for (*E*)-ethyl 3-(2-(2-oxo-1,2-dihydroquinolin-4-yl)hydrazono)propanoate (**8b**) (solvent omitted for clarity, displacement parameters are drawn at 30 % probability level).

**Fig. 5:**
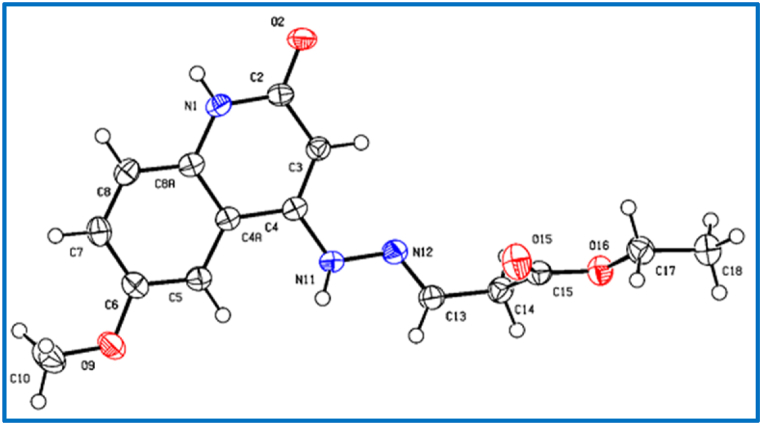
X-Ray crystallographic molecular structure for (*E*)-ethyl 3-(2-(6-methoxy-2-oxo-1,2-dihydroquinolin-4-yl)hydrazono)propanoate (**8c**) (displacement parameters are drawn at 50 % probability level).

**Fig. 6:**
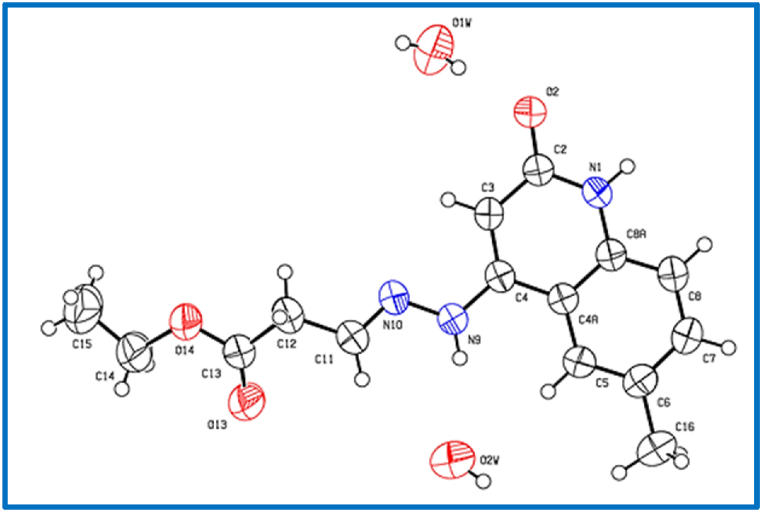
X-Ray crystallographic molecular structure for (*E*)-Ethyl 3-(2-(6-methyl-2-oxo-1,2-dihydroquinolin-4-yl)hydrazono)propanoate (**8d**) (solvent omitted for clarity, displacement parameters are drawn at 30 % probability level).

The single crystals, suitable for X-ray measurements, were obtained from methanol. X-ray crystal structure analysis were performed for compound **8b**, **8c** and **8d** (see Fig. 4, Fig. 5, Fig. 6), which indicate that our obtained products as *E*-isomer as shown in Fig. 3, Fig. 4, Fig. 5. Geometrical parameters are in the expected range for 1,2-dihydroquinolin-4-yl)hydrazono)propanoate (see Table 1). The 1,2dihydroquinoline and the hydrazono moieties are slightly twisted (7.4° (**8b**), 15.5° (**8c**), 8.3° (**8d**), see Table 1].

**Table 1:** Selected bond distances [Å], bond angles [°] and dihedral angles [°] for **8b**, **8c** and **8d** [in brackets for **8c**].

[in brackets for 8c]	8b	8c	8d
N1–C2	1.362(2)	1.3635(18)	1.354(2)
N1–H1	0.86	0.870(15)	0.86
C2–O2	1.259(2)	1.2628(17)	1.261(2)
C2–C3	1.421(2)	1.4255(19)	1.423(3)
C3–C4	1.364(2)	1.3627(19)	1.371(3)
C4–C4a	1.455(2)	1.4558(19)	1.445(2)
C4a-C8a	1.407)2)	1.411(2)	1.404(3)
N1–C8a	1.381(2)	1.3816(19)	1.381(3)
C4–N9 [C4–N11]	1.366(2)	1.3824(18)	1.377(2)
N9–N10 [N11–N12]	1.375(2)	1.3753(16)	1.366(2)
N9–H9 [N11–H11]	0.86	0.876(14)	0.86
N10–C11 [N12–C13]	1.261(2)	1.2659(19)	1.263(3)
C11–C12 [C13–C14]	1.490(2)	1.4966(19)	1.494(3)
C4–N9–N10 [C4–N11–N12]	118.85(14)	117.20(11)	118.25(15)
N9–N10–C11 [N11–N12–C13]	117.38(15)	115.86(12)	117.45(17)
N10–C11–C12 [N12–C13–C14]	119.44(17)	118.54(13)	118.47(19)
C3–C4–N9–N10 [C3–C4–N11–N12]	−1.8(3)	−1.1(2)	−5.0(3)
C4a-C4-N9-N10 [C4a-C4-N11-N12]	177.51(15)	178.63(11)	174.41(17)
C4–N9–N10–C11 [C4–N11–N12–C13}	−169.71(18)	156.97(13)	−170.8(2)
N9–N10–C11–C12 [N11–N12–C13–C14]	−179.37(17)	179.67(12)	−179.88(19)
angle between the normal of the l.S. planes of the 1,2dihydroquinoline and the hydrazono moiety [°]	7.4	15.5	8.3

The X-ray data of compound **8c** proves that (*E*)-ethyl 3-(2-(6-methyl-2-oxo-1,2-dihydroquinolin-4-yl)hydrazono)propanoate was formed exclusively from the reaction of **8c** with ethyl propiolate. All the X-ray structure confirmed the *E*-configuration concerning N10=C11 (**8b**, **8d**), N12=C13 (**8c**). As outlined from the X-ray structure, the configuration of all compounds in the *E*-form shows that pyrazole does not form because the *E*-form makes the electrophilic site far from the attack of the nucleophilic hydrazine-N^1^.

The crystal packing of **8b**, **8c** and **8d** shows intermolecular hydrogen bonds as well as π-π interactions.

### 3.1 Intermolecular hydrogen bonds for 8b, 8c and 8d

All three compounds form centrosymmetric dimers *via* intermolecular hydrogen bonds (N1–H1⋯O2). There are additional intermolecular hydrogen bond for **8b** and **8d** with the disordered solvent (MeOH for **8b** and water for **8d**) for N9–H9⋯O(solvent) and O–H(solvent)…O13. In addition, **8b** and **8d** are intermolecular hydrogen bonds *via* the ordered water molecule (O1W on special position at the 2-fold axis, O1W–H1W⋯O2) linking the dimers. For **8c**, there are additional intermolecular hydrogen bonds C13–H13⋯O2, N11–H11⋯O2 (see details below) [[33], [34], [35]].

### 3.2 Hydrogen bonds 8b

**Fig. 7:**
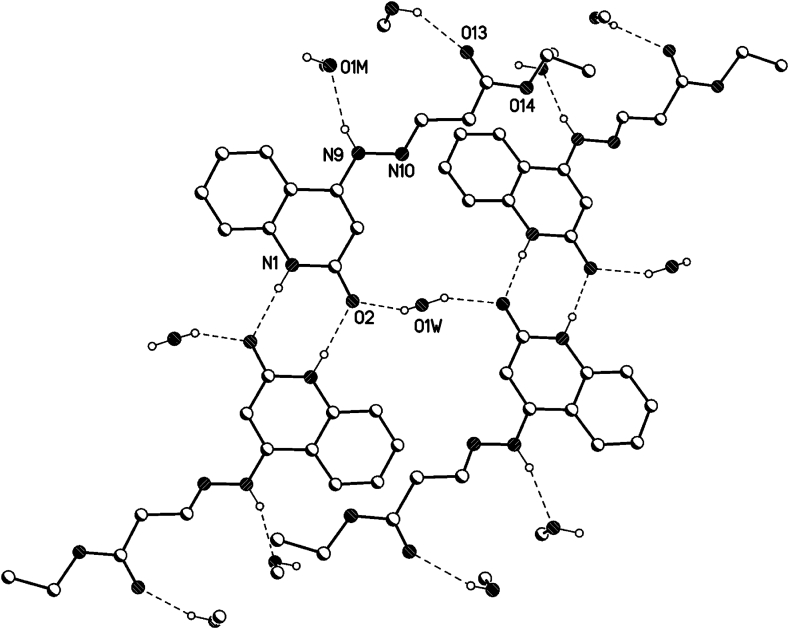
Intermolecular hydrogen bonds of compound **8b** showing hydrogen bonds as dashed lines.

**Fig. 8:**
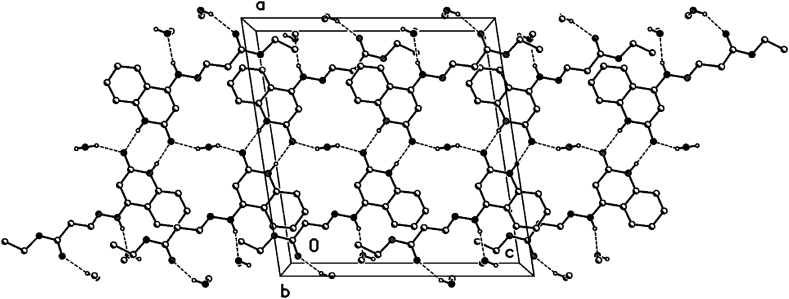
Crystal packing of compound **8b** showing hydrogen bonds as dashed lines.

**Fig. 9:**
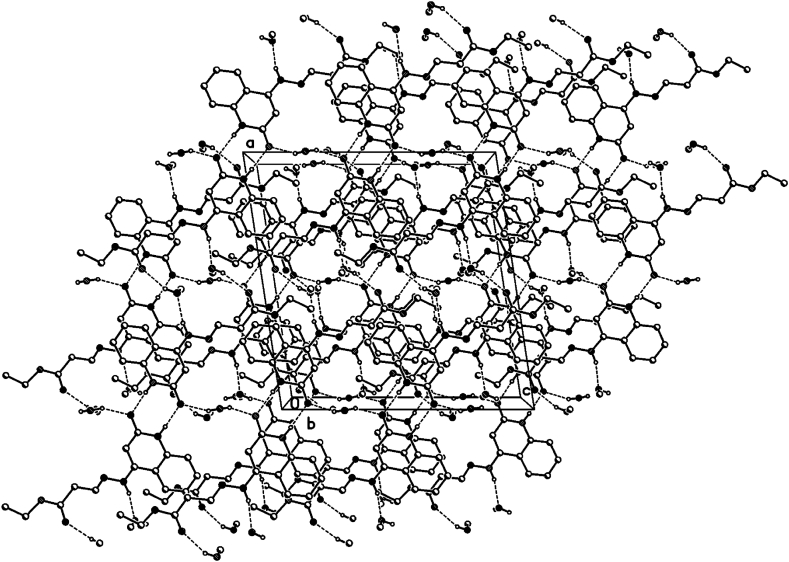
Crystal packing of compound **8b** showing hydrogen bonds as dashed lines.

In the crystal packing of **8b** (Fig. 7, Fig. 8, Fig. 9), the molecules are linked by intermolecular N9–H9⋯O1M, O1M-H1M···O13, O1W–H1W⋯O2, and N1–H1⋯O2 hydrogen bonds (Table 2) in a two-dimensional network parallel to the ac plane. The MeOH solvent molecule is disordered about a 2-fold axis (symmetry operator 1-x, y, 1.5-z), there is half a MeOH molecule in the asymmetric unit (or 0.5 MeOH molecule per formula moiety).

**Table 2:** Selected hydrogen-bond geometry (Å, °) for **8b**.

*D*—H···*A*	*D*—H	H···*A*	*D*···*A*	*D*—H···*A*
N1–H1⋯O2^i^	0.86	1.98	2.836 (2)	175
N9–H9⋯O1*M*^ii^	0.86	2.07	2.900 (3)	162
O1*M*—H1*M*···O13^iii^	0.82	2.19	2.823 (3)	135
O1*W*–H1*W*⋯O2	0.82 (1)	1.93 (3)	2.699 (2)	155 (5)

Symmetry codes: (i) -*x*+1, -*y*+1, -*z*+2; (ii) -*x*+1/2, *y*+1/2, -*z*+3/2; (iii) *x*+1/2, *y*-1/2, *z*.

### 3.3 Hydrogen bonds 8d

**Fig. 10:**
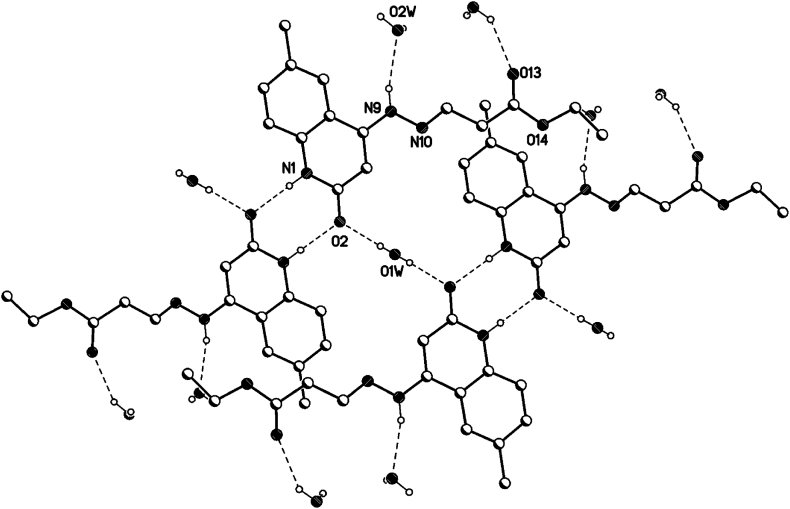
Intermolecular hydrogen bonds of compound **8d** showing hydrogen bonds as dashed lines.

**Fig. 11:**
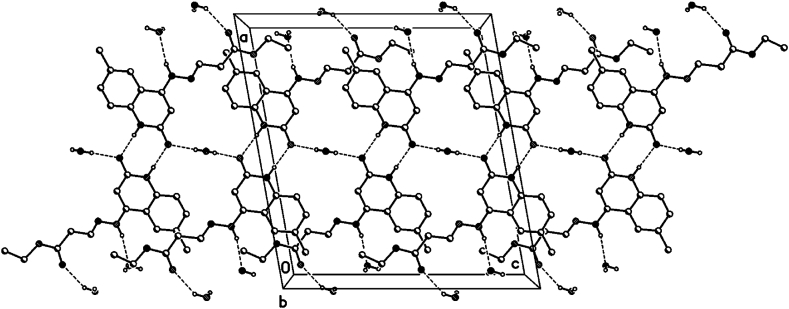
Crystal packing of compound **8d** showing hydrogen bonds as dashed lines.

**Fig. 12:**
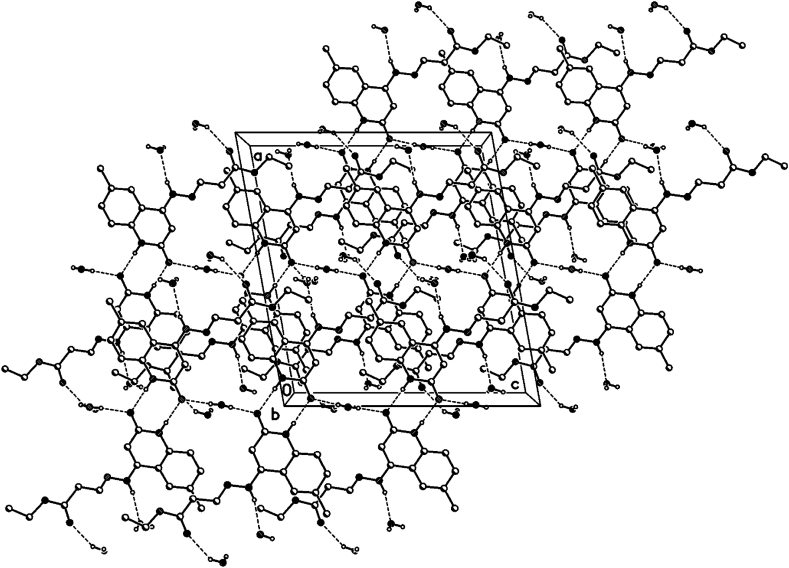
Crystal packing of compound **8d** showing hydrogen bonds as dashed lines.

In the crystal packing of **8d** (Fig. 10, Fig. 11, Fig. 12), the molecules are linked by intermolecular N9–H9⋯O2W, O2W–H2W1⋯O13, O1W–H1W⋯O2, and N1–H1⋯O2 hydrogen bonds (Table 3) in a two-dimensional network parallel to the ac plane. In 4 voids are 8 solvent water molecules (2 per void generated about a 2-fold axis (symmetry operator -x, y, 1.5-z, one water molecule per formula moiety). These water molecules are probably disordered.

**Table 3:** Selected hydrogen-bond geometry (Å, °) for **8d**.

*D*—H···*A*	*D*—H	H···*A*	*D*···*A*	*D*—H···*A*
N1–H1⋯O2^i^	0.86	1.98	2.839 (2)	178
N9–H9⋯O2*W*	0.86	2.20	3.044 (3)	165
O1*W*–H1*W*⋯O2	0.83 (1)	1.94 (2)	2.736 (2)	163 (5)
O2*W*–H2*W*1⋯O13^ii^	0.82 (1)	2.20 (5)	2.914 (3)	146 (7)

Symmetry codes: (i) -*x*+1, -*y*+1, -*z*+2; (ii) -*x*, *y*, -*z*+3/2.

### 3.4 Hydrogen bonds 8c

**Fig. 13:**
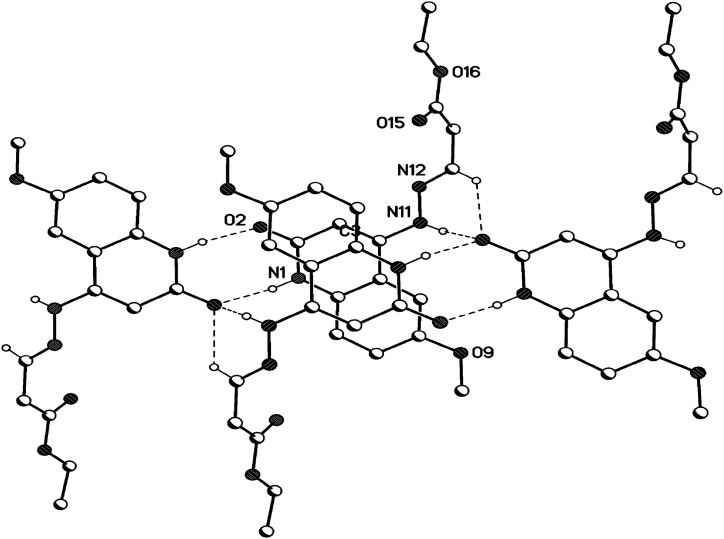
Intermolecular hydrogen bonds of compound **8c** showing hydrogen bonds as dashed lines.

**Fig. 14:**
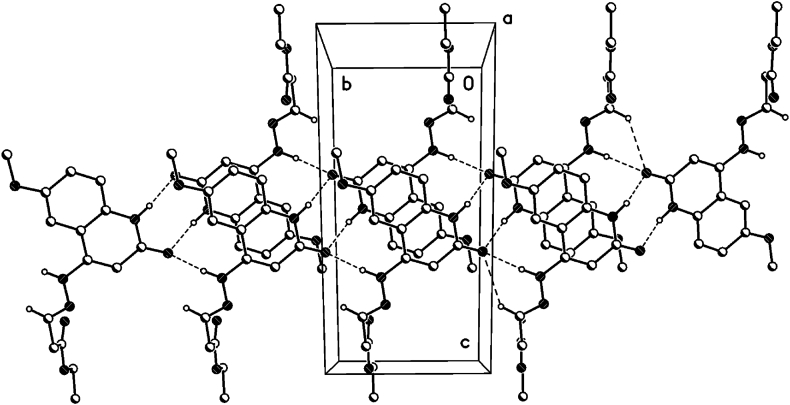
Crystal packing of compound **8c** showing hydrogen bonds as dashed lines.

**Fig. 15:**
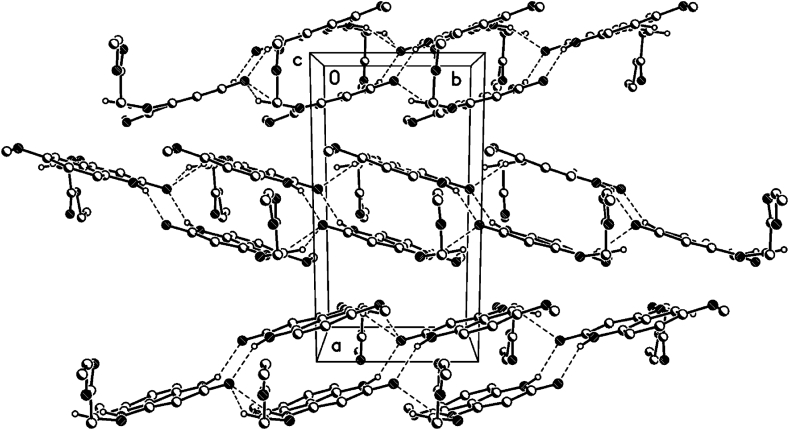
Crystal packing of compound **8c** showing hydrogen bonds as dashed lines.

In the crystal packing of **8c** (Fig. 13, Fig. 14, Fig. 15), the molecules are linked by intermolecular C13–H13⋯O2, N11–H11⋯O2, and N1–H1⋯O2 hydrogen bonds (Table 4) There are dimers in the b/c plane which are linked by bifurcular hydrogen bonds(C13–H13⋯O2, N11–H11⋯O2) forming an one-dimensional network along the a-axis, as well as π···π interactions along the a-axis (see below).

**Table 4:** Selected hydrogen-bond geometry (Å, °) for **8c**.

*D*—H···*A*	*D*—H	H···*A*	*D*···*A*	*D*—H···*A*
N1–H1⋯O2^i^	0.87 (2)	2.01 (2)	2.8219 (16)	154 (2)
N11–H11⋯O2^ii^	0.88 (1)	2.08 (2)	2.9003 (16)	155 (2)
C13–H13⋯O2^ii^	0.95	2.60	3.3397 (18)	135

Symmetry codes: (i) -*x*+1, -*y*+2, -*z*+1; (ii) *x*, *y*-1, *z*.

### 3.5 π···π interactions

**Fig. 16:**
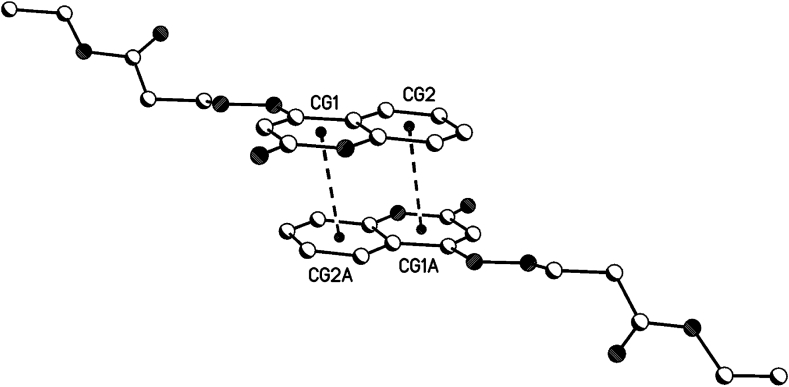
π···π interactions of compound **8b** (solvent and hydrogen atoms omitted for clarity).

**Fig. 17:**
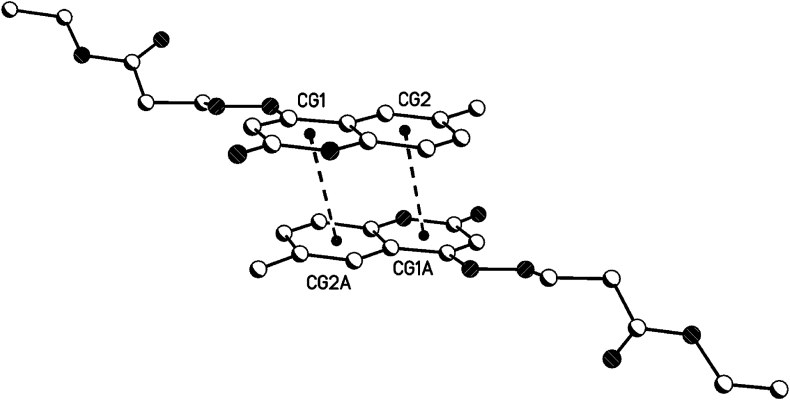
π···π interactions of compound **8d** (solvent and hydrogen atoms omitted for clarity).

**Fig. 18:**
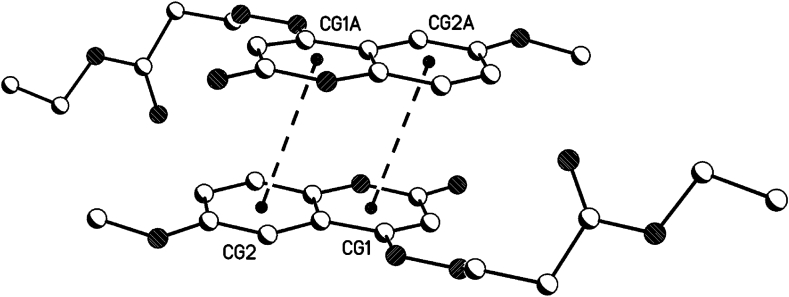
π···π interactions of compound **8c** (solvent and hydrogen atoms omitted for clarity).

For 1,2-dihydroquinoline moieties in all 3 compounds **8b**, **8c** and **8d**, centrosymmetric dimers are generated by π···π interactions (see Table 5, Fig. 16, Fig. 17, Fig. 18). In **8b** and 8d, these dimers linked the 2D-hydrogen bond networks in the a/c plane by π···π interactions in the direction of the b-axis. In **8c**, these dimers linked the hydrogen bonded dimers in the b/c plane by π···π interactions in the direction of the a-axis (see also the 1D hydrogen bond network along the a-axis).

**Table 5:** Geometrical parameters for the π-stacking moieties involved in the π···π interactions [Å, °] (Cg1 centroid of the ring N1–C2–C3–C4–C4a-C8a, Cg2 centroid of the ring C4a-C5-C6-C7-C8-C8a).

Compound	8b	8d	8c
centroid distance between Cg1…C2ga/Cg2…Cg1a	4.04	4.38	3.50
Vertical distance from ring centroids Cg1/Cg2 to symmetry related 1,2-dihydroquinolin	3.42	3.52	3.38
angle between the centroid vector Cg1…C2ga/Cg2…Cg1a and the normal to the 1,2-dihydroquinolin plane	147.9 (32.1)	143.3 (36.7)	165.0 (25.0)
symmetry operator (a)	0.5-x, 1.5-y, 2-z	0.5-x, 1.5-y, 2-z	1-x, 1-y, 1-z

The symmetry related planes of the 1,2-dihydroquinolin dimers are coplanar (centrosymmetric symmetry operator, see Table 5). Therefore, the angel between the symmetry related 1,2-dihydroquinolins are 0°.

**Scheme 3:**
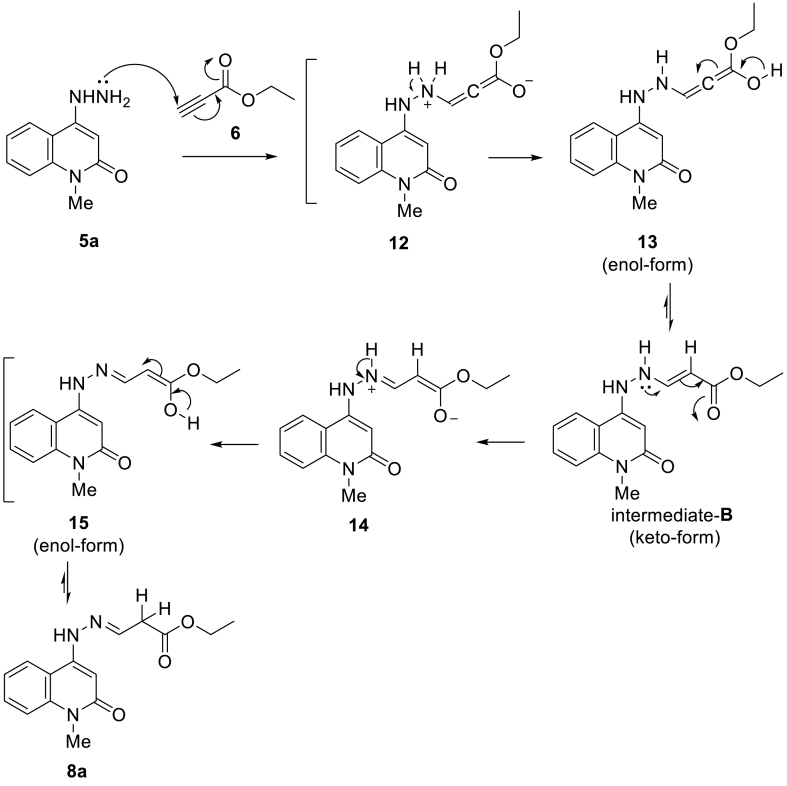
Postulated mechanism for the formation of compound **8a** ((*E*)-1-methyl-4-(2-(3-oxohexylidene)hydrazinyl)quinolin-2(1*H*)-one).

Based on the (*E*)-configuration of the products as attempted by X-ray crystallographic determination, the plausible mechanism of the nucleophilic addition reaction (1,4-addition) which is mentioned as aza-Michael addition as mentioned in Scheme 3. The mechanism involved two main nucleophilic additional steps; on each step, the nucleophilic attack occurs *via* the lone pairs of electrons on the hydrazine nitrogen (N^2^), and the final product was obtained through the formation of intermediates **12–15**. The steps of the mechanism involved two transformation of *enol*-form to *keto*-form structures: intermediate **13** (enol-form) to intermediate-**B** (keto-form) and intermediate **15** (enol-form) to the final product **8a** as the more stable keto-form.

## 4 Conclusion

We have reported in this article the synthesis of ethyl hydrazonoquinolone propanoate derivatives *via* a convenient reaction between substituted hydrazinyl-quinolinones and ethyl propiolate and determined the structure using advanced techniques of analyses and confirmed the correct structure using X-ray crystallographic analysis. Also, the X-ray discussion of the hydrogen bond among the molecules, as well as the pi-pi stacking interaction in the crystallatice.

## Data availability statement

Data associated with the study has not been deposited into a publicly available repository. Data will be made available on request.

## CRediT authorship contribution statement

**Hendawy N. Tawfeek:** Writing – original draft, Methodology, Conceptualization. **Ahmed M. Tawfeek:** Project administration. **Stefan Bräse:** Writing – review & editing, Formal analysis. **Martin Nieger:** Writing – review & editing, Formal analysis, Data curation. **Essmat M. El-Sheref:** Writing – review & editing.

## Declaration of competing interest

No conflict of interest exists.

All authors confirm that there are no known conflicts of interest associated with this publication and there has been no significant financial support for this work that could have influenced its outcome.

Dr. Hendawy N. Tawfeek (Corresponding author) hendawy1976@yahoo.com.
