# Supplementary material for: Stereoselective synthesis and X-ray structure determination of novel 1,2-dihydroquinolinehydrazonopropanoate derivatives
Source: Heliyon. 2024 Feb 1;10(4):e25248. doi: 10.1016/j.heliyon.2024.e25248 (PMC10884343; doi:10.1016/j.heliyon.2024.e25248)
Supplement: Multimedia component 1 [file mmc1.docx]

**Supplementary information**

Stereoselective synthesis and X-ray structure determination of novel 1,2-dihydroquinoline- hydrazonopropanoate derivatives

**Hendawy N. Tawfeeka,*, Ahmed M. Tawfeekb, Stefan Bräsec,*, Martin Niegerd,Essmat M. El-Sherefa**

a Chemistry Department, Faculty of Science, Minia University, El-Minia 61519, Egypt

b Chemistry Department, College of Science, King Saud University, Riyadh 11451, Saudi Arabia

c Institute of Biological and Chemical Systems, IBCS-FMS, Karlsruhe Institute of Technology, 76131 Karlsruhe, Germany

e Department of Chemistry, University of Helsinki, PO Box 55, A. I. Virtasen aukio 1, 00014 Helsinki, Finland

_______________________________________________________________________________________________________

* Correspondence: author: **Hendawy, N. Tawfeek**; E-mail: [hendawy1976@yahoo.com](mailto:hendawy1976@yahoo.com), ORCID: https://orcid.org/ 0000-0001-5943-4047. Chemistry Department, Faculty of Science, Minia University, El-Minia 61519, Egypt

*****Correspondence: author: **S. Bräse****;** Institute of Biological and Chemical Systems, IBCS-FMS, Karlsruhe Institute of Technology, 76131 Karlsruhe, Germany.E-mail: [braese@kit.edu](mailto:braese@kit.edu)

*Single crystal X-ray structure determination of* **8b**, **8c** and **8d**.

The single-crystal X-ray diffraction study was carried out on a Bruker D8 Venture diffractometer with PhotonII detector at 173(2) K or 298(2) K using Cu-Kα radiation (*λ* = 1.54178 Å). Dual space methods (SHELXT) [G. M. Sheldrick, *Acta Crystallogr.* 2015, **A71**, 3-8] were used for structure solution and refinement was carried out using SHELXL-2014 (full-matrix least-squares on *F2*) [G. M. Sheldrick, *Acta Crystallogr.* 2015, **C71**, 3-8]. Hydrogen atoms were refined using a riding model (H(O) free, except MeOH in **8b**). Semi-empirical absorption corrections were applied. For **8b** an extinction correction was applied.

*Compound* **8b (SB1463_HY_HA428)**

C14H15N3O3·0.5(CH4O)·0.5(H2O), Mr = 298.32 g mol-1, blocks yellow, size = 0.12 × 0.06 × 0.04 mm, monoclinic, *C2*/*c* (no.15), *a* = 16.981 (6) Å, *b* = 11.048 (4) Å, *c* = 16.479 (5) Å, *β = 98.60 (2)°*, λ = 1.54178 Å, V = 3056.8 (18) Å3, Z = 8, Dcalcd = 1.296 Mg m-3, F(000) = 1264, μ = 0.80 mm-1, T= 298 K, 13838 measured reflections (2θmax = 144.2oº) 2991 independent reflections [Rint = 0.049], 210 parameters and 148 restraints, R1 [for 2495 reflections with *I* > 2σ(*I*)] = 0.056 wR2 (for all data) = 0.177, S = 1.05, largest diff. peak and hole = 0.27 e Å-3/-0.23 e Å-3.

*Compound* **8c** (**SB1494_HY_ HA432**)

C15H17N3O4, Mr = 303.32 g mol-1, plates yellow, size = 0.16 × 0.12 × 0.02 mm, monoclinic, space group *P21/*n (no.14), *a* = 14.6490 (9) Å, *b* = 7.0414 (5) Å, *c* = 15.5731 (10) Å, *β* = 115.737 (2)°, λ = 1.54178, Å V = 1447.00 (17) Å3, Z = 4, Dcalcd = 1.392 Mg m-3, F(000) = 640, μ = 0.86 mm-1, T= 173 K, 14391 measured reflections (2θmax = 144.4º), 2838 independent reflections [Rint = 0.056], 206 parameters, 2 restraints, R1 [for 2549 reflections with *I* > 2σ(*I*)] = 0.050, wR2 (for all data) = 0.144, S = 1.04, largest diff. peak and hole = 0.32 e Å-3/-0.32 e Å-3.

*Compound* **8d** (**SB1466_HY_HA427**)

C15H17N3O 1.5(H2O), Mr = 314.34, g mol-1, plates yellow, size = 0.35 0.25 × 0.20 mm, monoclinic, space group I (no.15), *a* = 17.8791 (3) Å, *b* = 11.1773 (2) Å, *c* = 16.4921 (2) Å, *β* = 100.178 (1)°, λ = 1.54178, Å V = 3243.92 (9) Å3, Z = 8, Dcalcd = 1.287 Mg m-3, F(000) = 1336, μ = 0.80 mm-1, T= 298 K, 16724 measured reflections (2θmax = 144.4º), 3185 independent reflections [Rint = 0.056], 214 parameters, 169 restraints, R1 [for 2799 reflections with *I* > 2σ(*I*)] = 0.069, wR2 (for all data) = 0.215, S = 1.08, largest diff. peak and hole = 0.42 e Å-3/-0.44 e Å-3.

**Supplementary data**

CCDC 2256765 (**8b**), 2256766 (**8c**), and 2256767 (**8d**) contain the supplementary crystallographic data for this paper. These data can be obtained free of charge from The Cambridge Crystallographic Data Centre via [www.ccdc.cam.ac.uk/data_request/cif](http://www.ccdc.cam.ac.uk/data_request/cif) (deposited at the Cambride Structural Database17.04.2023).





**Figure SI1.** X-Ray crystallographic molecular structure of compound **8b** ((*E*)-ethyl 3-(2-(2-oxo-1,2-dihydroquinolin-4-yl)hydrazono)propanoate) (solvent omitted, displacement parameters are drawn at 30% probability level)

*Crystal data for* ***8b***

| C14H15N3O3·0.5(CH4O)·0.5(H2O) | *F*(000) = 1264 |
| --- | --- |
| *Mr* = 298.32 | *D*x = 1.296 Mg m-3 |
| Monoclinic, *C*2/*c (no.15)* | Cu *K*α radiation,  = 1.54178 Å |
| *a* = 16.981 (6) Å | Cell parameters from 6626 reflections |
| *b* = 11.048 (4) Å |  = 4.7–71.8° |
| *c* = 16.479 (5) Å |  = 0.80 mm-1 |
| β = 98.60 (2)° | *T* = 298 K |
| *V* = 3056.8 (18) Å3 | Blocks, yellow |
| *Z* = 8 | 0.12 × 0.06 × 0.04 mm |

*Data collection for* ***8b***

| Bruker D8 VENTURE diffractometer with PhotonII CPAD detector | 2495 reflections with *I* > 2σ(*I*) |
| --- | --- |
| Radiation source: INCOATEC microfocus sealed tube | *R*int = 0.049 |
| rotation in  and , 1°, shutterless scans | max = 72.1°, min = 4.8° |
| Absorption correction: multi-scan  *SADABS* (Sheldrick, 2014) | *h* = -1620 |
| *T*min = 0.689, *T*max = 0.971 | *k* = -1213 |
| 13838 measured reflections | *l* = -2020 |
| 2991 independent reflections |  |

*Refinement for* ***8b***

| Refinement on *F*2 | Secondary atom site location: difference Fourier map |
| --- | --- |
| Least-squares matrix: full | Hydrogen site location: mixed |
| *R*[*F*2 > 2σ(*F*2)] = 0.056 | H atoms treated by a mixture of independent and constrained refinement |
| *wR*(*F*2) = 0.177 | *w* = 1/[2(*F*o2) + (0.1006*P*)2 + 1.0772*P*]  where *P* = (*F*o2 + 2*F*c2)/3 |
| *S* = 1.05 | (Δ/σ)max < 0.001 |
| 2991 reflections | max = 0.27 e Å-3 |
| 210 parameters | min = -0.23 e Å-3 |
| 148 restraints | Extinction correction: *SHELXL2014*/7 (Sheldrick 2014), Fc*=kFc[1+0.001xFc2λ3/sin(2θ)]-1/4 |
| Primary atom site location: dual | Extinction coefficient: 0.0010 (2) |

*Fractional atomic coordinates and isotropic or equivalent isotropic displacement parameters (Å2) for* ***8b***

|  | *x* | *y* | *z* | *U*iso*/*U*eq | Occ. (<1) |
| --- | --- | --- | --- | --- | --- |
| N1 | 0.39305 (8) | 0.54368 (15) | 0.98880 (8) | 0.0596 (4) |  |
| H1 | 0.4319 | 0.5236 | 1.0260 | 0.072* |  |
| C2 | 0.40755 (10) | 0.55430 (17) | 0.91003 (9) | 0.0576 (4) |  |
| O2 | 0.47675 (8) | 0.53395 (16) | 0.89463 (7) | 0.0767 (5) |  |
| C3 | 0.34285 (10) | 0.58911 (16) | 0.84962 (9) | 0.0559 (4) |  |
| H3 | 0.3511 | 0.5963 | 0.7953 | 0.067* |  |
| C4 | 0.26899 (10) | 0.61226 (15) | 0.86906 (9) | 0.0524 (4) |  |
| C4A | 0.25436 (10) | 0.59657 (15) | 0.95322 (9) | 0.0531 (4) |  |
| C5 | 0.18059 (11) | 0.61532 (18) | 0.97922 (11) | 0.0634 (5) |  |
| H5 | 0.1367 | 0.6366 | 0.9411 | 0.076* |  |
| C6 | 0.17207 (12) | 0.6026 (2) | 1.06082 (12) | 0.0702 (5) |  |
| H6 | 0.1226 | 0.6150 | 1.0773 | 0.084* |  |
| C7 | 0.23722 (13) | 0.57130 (19) | 1.11828 (12) | 0.0701 (5) |  |
| H7 | 0.2314 | 0.5641 | 1.1733 | 0.084* |  |
| C8 | 0.30992 (12) | 0.55092 (19) | 1.09470 (11) | 0.0656 (5) |  |
| H8 | 0.3531 | 0.5288 | 1.1335 | 0.079* |  |
| C8A | 0.31937 (10) | 0.56332 (16) | 1.01227 (9) | 0.0541 (4) |  |
| N9 | 0.20772 (8) | 0.65135 (15) | 0.81185 (8) | 0.0624 (4) |  |
| H9 | 0.1612 | 0.6635 | 0.8250 | 0.075* |  |
| N10 | 0.22165 (8) | 0.67101 (14) | 0.73283 (8) | 0.0576 (4) |  |
| C11 | 0.16788 (11) | 0.7238 (2) | 0.68454 (11) | 0.0678 (5) |  |
| H11 | 0.1212 | 0.7486 | 0.7028 | 0.081* |  |
| C12 | 0.17975 (11) | 0.7461 (2) | 0.59800 (11) | 0.0675 (5) |  |
| H12A | 0.2270 | 0.7030 | 0.5876 | 0.081* |  |
| H12B | 0.1891 | 0.8318 | 0.5911 | 0.081* |  |
| C13 | 0.11043 (11) | 0.7073 (2) | 0.53542 (11) | 0.0670 (5) |  |
| O13 | 0.04581 (9) | 0.6798 (2) | 0.54980 (9) | 0.1021 (6) |  |
| O14 | 0.12966 (8) | 0.70731 (16) | 0.46098 (8) | 0.0780 (5) |  |
| C14 | 0.06853 (13) | 0.6710 (3) | 0.39370 (13) | 0.0909 (7) |  |
| H14A | 0.0257 | 0.7300 | 0.3862 | 0.109* |  |
| H14B | 0.0465 | 0.5930 | 0.4053 | 0.109* |  |
| C15 | 0.10712 (18) | 0.6637 (5) | 0.31880 (15) | 0.1318 (14) |  |
| H15A | 0.0684 | 0.6399 | 0.2730 | 0.198* |  |
| H15B | 0.1286 | 0.7414 | 0.3080 | 0.198* |  |
| H15C | 0.1493 | 0.6050 | 0.3269 | 0.198* |  |
| O1M | 0.45355 (16) | 0.2266 (4) | 0.67535 (18) | 0.0848 (9) | 0.5 |
| H1M | 0.4700 | 0.2523 | 0.6343 | 0.127* | 0.5 |
| C1M | 0.4683 (2) | 0.1178 (6) | 0.6829 (3) | 0.0830 (12) | 0.5 |
| H1M1 | 0.5075 | 0.1049 | 0.7304 | 0.125* | 0.5 |
| H1M2 | 0.4883 | 0.0889 | 0.6350 | 0.125* | 0.5 |
| H1M3 | 0.4205 | 0.0747 | 0.6892 | 0.125* | 0.5 |
| O1W | 0.5000 | 0.4361 (3) | 0.7500 | 0.1245 (11) |  |
| H1W | 0.506 (3) | 0.475 (4) | 0.7928 (18) | 0.187* |  |

*Atomic displacement parameters (Å2) for* ***8b***

|  | *U*11 | *U*22 | *U*33 | *U*12 | *U*13 | *U*23 |
| --- | --- | --- | --- | --- | --- | --- |
| N1 | 0.0551 (8) | 0.0833 (10) | 0.0397 (7) | 0.0076 (7) | 0.0048 (5) | 0.0083 (6) |
| C2 | 0.0557 (9) | 0.0745 (11) | 0.0429 (8) | 0.0056 (8) | 0.0080 (6) | 0.0049 (7) |
| O2 | 0.0567 (7) | 0.1279 (12) | 0.0463 (6) | 0.0195 (7) | 0.0102 (5) | 0.0134 (7) |
| C3 | 0.0583 (9) | 0.0702 (10) | 0.0391 (7) | 0.0079 (8) | 0.0071 (6) | 0.0049 (7) |
| C4 | 0.0545 (8) | 0.0593 (9) | 0.0423 (8) | 0.0037 (7) | 0.0042 (6) | -0.0009 (6) |
| C4A | 0.0574 (9) | 0.0568 (9) | 0.0456 (8) | -0.0009 (7) | 0.0091 (6) | -0.0009 (6) |
| C5 | 0.0602 (10) | 0.0724 (11) | 0.0589 (9) | 0.0022 (8) | 0.0136 (7) | -0.0014 (8) |
| C6 | 0.0715 (11) | 0.0778 (12) | 0.0672 (11) | -0.0009 (10) | 0.0292 (9) | 0.0002 (9) |
| C7 | 0.0885 (13) | 0.0746 (12) | 0.0526 (9) | 0.0002 (10) | 0.0277 (9) | 0.0057 (8) |
| C8 | 0.0761 (12) | 0.0766 (12) | 0.0454 (9) | 0.0022 (9) | 0.0130 (8) | 0.0083 (7) |
| C8A | 0.0594 (9) | 0.0592 (9) | 0.0442 (8) | -0.0005 (7) | 0.0100 (7) | 0.0024 (6) |
| N9 | 0.0542 (8) | 0.0880 (11) | 0.0449 (7) | 0.0126 (7) | 0.0067 (6) | 0.0018 (6) |
| N10 | 0.0580 (8) | 0.0703 (9) | 0.0433 (7) | 0.0097 (7) | 0.0035 (5) | 0.0002 (6) |
| C11 | 0.0584 (10) | 0.0908 (13) | 0.0527 (9) | 0.0177 (9) | 0.0036 (7) | 0.0039 (8) |
| C12 | 0.0665 (11) | 0.0815 (12) | 0.0521 (10) | 0.0099 (9) | 0.0011 (8) | 0.0102 (8) |
| C13 | 0.0586 (10) | 0.0872 (13) | 0.0543 (9) | 0.0135 (9) | 0.0059 (7) | 0.0063 (8) |
| O13 | 0.0676 (9) | 0.1709 (19) | 0.0694 (9) | -0.0074 (10) | 0.0158 (7) | -0.0140 (10) |
| O14 | 0.0640 (8) | 0.1190 (12) | 0.0491 (7) | 0.0006 (7) | 0.0018 (6) | 0.0065 (7) |
| C14 | 0.0671 (12) | 0.142 (2) | 0.0594 (11) | 0.0011 (13) | -0.0052 (9) | -0.0038 (12) |
| C15 | 0.0948 (18) | 0.240 (5) | 0.0573 (13) | -0.017 (2) | 0.0020 (12) | -0.0016 (18) |
| O1M | 0.0484 (14) | 0.138 (3) | 0.0703 (17) | -0.0095 (17) | 0.0153 (12) | -0.0036 (18) |
| C1M | 0.053 (2) | 0.128 (4) | 0.070 (2) | -0.021 (2) | 0.0154 (18) | -0.017 (2) |
| O1W | 0.182 (3) | 0.131 (2) | 0.0688 (15) | 0.000 | 0.0452 (19) | 0.000 |

*Geometric parameters (Å, º) for* ***8b***

| N1—C2 | 1.362 (2) | N10—C11 | 1.261 (2) |
| --- | --- | --- | --- |
| N1—C8A | 1.381 (2) | C11—C12 | 1.490 (3) |
| N1—H1 | 0.8600 | C11—H11 | 0.9300 |
| C2—O2 | 1.259 (2) | C12—C13 | 1.506 (3) |
| C2—C3 | 1.421 (2) | C12—H12A | 0.9700 |
| C3—C4 | 1.364 (2) | C12—H12B | 0.9700 |
| C3—H3 | 0.9300 | C13—O13 | 1.196 (3) |
| C4—N9 | 1.366 (2) | C13—O14 | 1.316 (2) |
| C4—C4A | 1.455 (2) | O14—C14 | 1.457 (2) |
| C4A—C5 | 1.399 (2) | C14—C15 | 1.484 (3) |
| C4A—C8A | 1.407 (2) | C14—H14A | 0.9700 |
| C5—C6 | 1.381 (3) | C14—H14B | 0.9700 |
| C5—H5 | 0.9300 | C15—H15A | 0.9600 |
| C6—C7 | 1.388 (3) | C15—H15B | 0.9600 |
| C6—H6 | 0.9300 | C15—H15C | 0.9600 |
| C7—C8 | 1.367 (3) | O1M—C1M | 1.230 (6) |
| C7—H7 | 0.9300 | O1M—H1M | 0.8200 |
| C8—C8A | 1.398 (2) | C1M—H1M1 | 0.9600 |
| C8—H8 | 0.9300 | C1M—H1M2 | 0.9600 |
| N9—N10 | 1.3751 (19) | C1M—H1M3 | 0.9600 |
| N9—H9 | 0.8600 | O1W—H1W | 0.820 (10) |
|  |  |  |  |
| C2—N1—C8A | 123.64 (14) | C11—N10—N9 | 117.38 (15) |
| C2—N1—H1 | 118.2 | N10—C11—C12 | 119.44 (17) |
| C8A—N1—H1 | 118.2 | N10—C11—H11 | 120.3 |
| O2—C2—N1 | 118.95 (15) | C12—C11—H11 | 120.3 |
| O2—C2—C3 | 123.67 (15) | C11—C12—C13 | 113.85 (17) |
| N1—C2—C3 | 117.38 (15) | C11—C12—H12A | 108.8 |
| C4—C3—C2 | 121.86 (14) | C13—C12—H12A | 108.8 |
| C4—C3—H3 | 119.1 | C11—C12—H12B | 108.8 |
| C2—C3—H3 | 119.1 | C13—C12—H12B | 108.8 |
| C3—C4—N9 | 121.97 (14) | H12A—C12—H12B | 107.7 |
| C3—C4—C4A | 119.70 (14) | O13—C13—O14 | 123.20 (18) |
| N9—C4—C4A | 118.33 (14) | O13—C13—C12 | 125.65 (18) |
| C5—C4A—C8A | 118.22 (15) | O14—C13—C12 | 111.14 (16) |
| C5—C4A—C4 | 124.24 (16) | C13—O14—C14 | 117.49 (16) |
| C8A—C4A—C4 | 117.52 (15) | O14—C14—C15 | 107.16 (19) |
| C6—C5—C4A | 120.82 (18) | O14—C14—H14A | 110.3 |
| C6—C5—H5 | 119.6 | C15—C14—H14A | 110.3 |
| C4A—C5—H5 | 119.6 | O14—C14—H14B | 110.3 |
| C5—C6—C7 | 120.04 (18) | C15—C14—H14B | 110.3 |
| C5—C6—H6 | 120.0 | H14A—C14—H14B | 108.5 |
| C7—C6—H6 | 120.0 | C14—C15—H15A | 109.5 |
| C8—C7—C6 | 120.57 (17) | C14—C15—H15B | 109.5 |
| C8—C7—H7 | 119.7 | H15A—C15—H15B | 109.5 |
| C6—C7—H7 | 119.7 | C14—C15—H15C | 109.5 |
| C7—C8—C8A | 119.97 (18) | H15A—C15—H15C | 109.5 |
| C7—C8—H8 | 120.0 | H15B—C15—H15C | 109.5 |
| C8A—C8—H8 | 120.0 | C1M—O1M—H1M | 109.5 |
| N1—C8A—C8 | 119.81 (16) | O1M—C1M—H1M1 | 109.5 |
| N1—C8A—C4A | 119.83 (14) | O1M—C1M—H1M2 | 109.5 |
| C8—C8A—C4A | 120.36 (16) | H1M1—C1M—H1M2 | 109.5 |
| C4—N9—N10 | 118.85 (14) | O1M—C1M—H1M3 | 109.5 |
| C4—N9—H9 | 120.6 | H1M1—C1M—H1M3 | 109.5 |
| N10—N9—H9 | 120.6 | H1M2—C1M—H1M3 | 109.5 |
|  |  |  |  |
| C8A—N1—C2—O2 | -179.67 (18) | C7—C8—C8A—N1 | 179.44 (18) |
| C8A—N1—C2—C3 | 0.8 (3) | C7—C8—C8A—C4A | -0.1 (3) |
| O2—C2—C3—C4 | -178.91 (19) | C5—C4A—C8A—N1 | 179.80 (16) |
| N1—C2—C3—C4 | 0.5 (3) | C4—C4A—C8A—N1 | -1.8 (3) |
| C2—C3—C4—N9 | 176.78 (17) | C5—C4A—C8A—C8 | -0.7 (3) |
| C2—C3—C4—C4A | -2.5 (3) | C4—C4A—C8A—C8 | 177.77 (16) |
| C3—C4—C4A—C5 | -178.59 (17) | C3—C4—N9—N10 | -1.8 (3) |
| N9—C4—C4A—C5 | 2.1 (3) | C4A—C4—N9—N10 | 177.51 (15) |
| C3—C4—C4A—C8A | 3.1 (3) | C4—N9—N10—C11 | -169.71 (18) |
| N9—C4—C4A—C8A | -176.24 (16) | N9—N10—C11—C12 | -179.37 (17) |
| C8A—C4A—C5—C6 | 0.6 (3) | N10—C11—C12—C13 | 130.7 (2) |
| C4—C4A—C5—C6 | -177.74 (17) | C11—C12—C13—O13 | 12.1 (3) |
| C4A—C5—C6—C7 | 0.3 (3) | C11—C12—C13—O14 | -167.94 (17) |
| C5—C6—C7—C8 | -1.0 (3) | O13—C13—O14—C14 | -0.4 (3) |
| C6—C7—C8—C8A | 1.0 (3) | C12—C13—O14—C14 | 179.7 (2) |
| C2—N1—C8A—C8 | -179.70 (17) | C13—O14—C14—C15 | -173.1 (3) |
| C2—N1—C8A—C4A | -0.2 (3) |  |  |

*Hydrogen-bond geometry (Å, º) for* ***8b***

| *D*—H···*A* | *D*—H | H···*A* | *D*···*A* | *D*—H···*A* |
| --- | --- | --- | --- | --- |
| N1—H1···O2i | 0.86 | 1.98 | 2.836 (2) | 175 |
| N9—H9···O1*M*ii | 0.86 | 2.07 | 2.900 (3) | 162 |
| O1*W*—H1*W*···O2 | 0.82 (1) | 1.93 (3) | 2.699 (2) | 155 (5) |

Symmetry codes: (i) -*x*+1, -*y*+1, -*z*+2; (ii) -*x*+1/2, *y*+1/2, -*z*+3/2.





**Figure SI2**. X-Ray crystallographic molecular structure of compound **8c** ((*E*)-ethyl 3-(2-(6-methoxy-2-oxo-1,2-dihydroquinolin-4-yl)hydrazono)propanoate) (displacement parameters are drawn at 50% probability level)

*Crystal data for* ***8c***

| C15H17N3O4 | *F*(000) = 640 |
| --- | --- |
| *Mr* = 303.32 | *D*x = 1.392 Mg m-3 |
| Monoclinic, *P*21/*n (no.14)* | Cu *K*α radiation,  = 1.54178 Å |
| *a* = 14.6490 (9) Å | Cell parameters from 8967 reflections |
| *b* = 7.0414 (5) Å |  = 3.4–72.1° |
| *c* = 15.5731 (10) Å |  = 0.86 mm-1 |
| β = 115.737 (2)° | *T* = 173 K |
| *V* = 1447.00 (17) Å3 | Plates, yellow |
| *Z* = 4 | 0.16 × 0.12 × 0.02 mm |

*Data collection for* ***8c***

| Bruker D8 VENTURE diffractometer with PhotonII CPAD detector | 2549 reflections with *I* > 2σ(*I*) |
| --- | --- |
| Radiation source: INCOATEC microfocus sealed tube | *R*int = 0.056 |
| rotation in  and , 1°, shutterless scans | max = 72.2°, min = 3.5° |
| Absorption correction: multi-scan  *SADABS* (Sheldrick, 2014) | *h* = -1817 |
| *T*min = 0.728, *T*max = 0.987 | *k* = -78 |
| 14391 measured reflections | *l* = -1919 |
| 2838 independent reflections |  |

*Refinement for* ***8c***

| Refinement on *F*2 | Primary atom site location: dual |
| --- | --- |
| Least-squares matrix: full | Secondary atom site location: difference Fourier map |
| *R*[*F*2 > 2σ(*F*2)] = 0.050 | Hydrogen site location: difference Fourier map |
| *wR*(*F*2) = 0.144 | H atoms treated by a mixture of independent and constrained refinement |
| *S* = 1.04 | *w* = 1/[2(*F*o2) + (0.0923*P*)2 + 0.3716*P*]  where *P* = (*F*o2 + 2*F*c2)/3 |
| 2838 reflections | (Δ/σ)max < 0.001 |
| 206 parameters | max = 0.32 e Å-3 |
| 2 restraints | min = -0.32 e Å-3 |

*Fractional atomic coordinates and isotropic or equivalent isotropic displacement parameters (Å2) for* ***8c***

|  | *x* | *y* | *z* | *U*iso*/*U*eq |
| --- | --- | --- | --- | --- |
| N1 | 0.41624 (9) | 0.78826 (17) | 0.48185 (8) | 0.0270 (3) |
| H1 | 0.4423 (13) | 0.877 (2) | 0.5243 (12) | 0.032* |
| C2 | 0.41490 (11) | 0.81796 (19) | 0.39479 (10) | 0.0266 (3) |
| O2 | 0.44199 (8) | 0.97670 (14) | 0.37592 (7) | 0.0326 (3) |
| C3 | 0.38600 (10) | 0.6622 (2) | 0.32987 (10) | 0.0260 (3) |
| H3 | 0.3826 | 0.6797 | 0.2681 | 0.031* |
| C4 | 0.36310 (10) | 0.48885 (19) | 0.35421 (10) | 0.0246 (3) |
| C4A | 0.35974 (10) | 0.46311 (19) | 0.44555 (10) | 0.0244 (3) |
| C5 | 0.32889 (11) | 0.2940 (2) | 0.47291 (10) | 0.0273 (3) |
| H5 | 0.3077 | 0.1895 | 0.4301 | 0.033* |
| C6 | 0.32892 (11) | 0.2777 (2) | 0.56133 (11) | 0.0299 (3) |
| C7 | 0.36055 (11) | 0.4315 (2) | 0.62525 (10) | 0.0318 (3) |
| H7 | 0.3613 | 0.4201 | 0.6864 | 0.038* |
| C8 | 0.39048 (11) | 0.5995 (2) | 0.59907 (10) | 0.0295 (3) |
| H8 | 0.4120 | 0.7030 | 0.6425 | 0.035* |
| C8A | 0.38940 (10) | 0.6185 (2) | 0.50907 (10) | 0.0257 (3) |
| O9 | 0.29557 (9) | 0.10712 (16) | 0.57936 (8) | 0.0374 (3) |
| C10 | 0.30522 (17) | 0.0725 (3) | 0.67279 (14) | 0.0535 (5) |
| H10A | 0.3769 | 0.0775 | 0.7183 | 0.080* |
| H10B | 0.2778 | -0.0533 | 0.6751 | 0.080* |
| H10C | 0.2676 | 0.1694 | 0.6894 | 0.080* |
| N11 | 0.34201 (9) | 0.33259 (16) | 0.29492 (8) | 0.0278 (3) |
| H11 | 0.3536 (14) | 0.217 (2) | 0.3176 (13) | 0.033* |
| N12 | 0.34315 (9) | 0.35871 (17) | 0.20787 (8) | 0.0293 (3) |
| C13 | 0.35803 (11) | 0.2111 (2) | 0.16930 (10) | 0.0293 (3) |
| H13 | 0.3680 | 0.0912 | 0.2000 | 0.035* |
| C14 | 0.35958 (12) | 0.2296 (2) | 0.07420 (11) | 0.0306 (3) |
| H14A | 0.3231 | 0.3467 | 0.0425 | 0.037* |
| H14B | 0.3236 | 0.1202 | 0.0337 | 0.037* |
| C15 | 0.46617 (11) | 0.2371 (2) | 0.08301 (11) | 0.0295 (3) |
| O15 | 0.54219 (9) | 0.24219 (18) | 0.15687 (8) | 0.0429 (3) |
| O16 | 0.46541 (8) | 0.24099 (16) | -0.00314 (7) | 0.0337 (3) |
| C17 | 0.56394 (12) | 0.2615 (2) | -0.00285 (12) | 0.0349 (4) |
| H17A | 0.6082 | 0.1534 | 0.0309 | 0.042* |
| H17B | 0.5968 | 0.3805 | 0.0299 | 0.042* |
| C18 | 0.54711 (14) | 0.2661 (3) | -0.10526 (12) | 0.0411 (4) |
| H18A | 0.6124 | 0.2800 | -0.1079 | 0.062* |
| H18B | 0.5147 | 0.1477 | -0.1368 | 0.062* |
| H18C | 0.5033 | 0.3738 | -0.1378 | 0.062* |

*Atomic displacement parameters (Å2) for* ***8c***

|  | *U*11 | *U*22 | *U*33 | *U*12 | *U*13 | *U*23 |
| --- | --- | --- | --- | --- | --- | --- |
| N1 | 0.0295 (6) | 0.0226 (6) | 0.0258 (6) | -0.0003 (5) | 0.0092 (5) | -0.0036 (5) |
| C2 | 0.0265 (7) | 0.0209 (6) | 0.0300 (7) | 0.0030 (5) | 0.0098 (6) | 0.0015 (5) |
| O2 | 0.0407 (6) | 0.0208 (5) | 0.0318 (5) | -0.0028 (4) | 0.0117 (5) | 0.0010 (4) |
| C3 | 0.0270 (7) | 0.0245 (7) | 0.0254 (7) | 0.0017 (5) | 0.0104 (6) | 0.0003 (5) |
| C4 | 0.0204 (6) | 0.0243 (7) | 0.0265 (7) | 0.0012 (5) | 0.0078 (5) | -0.0016 (5) |
| C4A | 0.0206 (6) | 0.0244 (7) | 0.0263 (7) | 0.0023 (5) | 0.0084 (5) | -0.0002 (5) |
| C5 | 0.0255 (7) | 0.0259 (7) | 0.0295 (7) | 0.0007 (5) | 0.0111 (6) | -0.0003 (5) |
| C6 | 0.0259 (7) | 0.0309 (7) | 0.0324 (8) | 0.0024 (6) | 0.0123 (6) | 0.0056 (6) |
| C7 | 0.0296 (7) | 0.0393 (8) | 0.0262 (7) | 0.0035 (6) | 0.0118 (6) | 0.0038 (6) |
| C8 | 0.0274 (7) | 0.0317 (7) | 0.0261 (7) | 0.0026 (6) | 0.0083 (6) | -0.0028 (5) |
| C8A | 0.0211 (6) | 0.0249 (7) | 0.0283 (7) | 0.0027 (5) | 0.0079 (5) | -0.0005 (5) |
| O9 | 0.0433 (7) | 0.0353 (6) | 0.0368 (6) | -0.0044 (5) | 0.0204 (5) | 0.0059 (5) |
| C10 | 0.0673 (13) | 0.0509 (11) | 0.0381 (9) | -0.0085 (9) | 0.0187 (9) | 0.0136 (8) |
| N11 | 0.0358 (7) | 0.0199 (6) | 0.0296 (6) | -0.0014 (5) | 0.0159 (5) | -0.0007 (5) |
| N12 | 0.0318 (6) | 0.0271 (6) | 0.0278 (6) | -0.0030 (5) | 0.0120 (5) | -0.0019 (5) |
| C13 | 0.0308 (7) | 0.0266 (7) | 0.0299 (7) | -0.0010 (6) | 0.0126 (6) | -0.0009 (5) |
| C14 | 0.0310 (8) | 0.0313 (7) | 0.0290 (7) | -0.0011 (6) | 0.0125 (6) | -0.0024 (6) |
| C15 | 0.0313 (8) | 0.0257 (7) | 0.0299 (7) | 0.0000 (5) | 0.0118 (6) | -0.0001 (5) |
| O15 | 0.0320 (6) | 0.0604 (8) | 0.0318 (6) | -0.0004 (5) | 0.0097 (5) | 0.0026 (5) |
| O16 | 0.0303 (6) | 0.0409 (6) | 0.0301 (6) | -0.0020 (4) | 0.0133 (5) | -0.0013 (4) |
| C17 | 0.0314 (8) | 0.0374 (8) | 0.0380 (8) | -0.0017 (6) | 0.0170 (7) | 0.0003 (6) |
| C18 | 0.0453 (10) | 0.0441 (9) | 0.0389 (9) | -0.0008 (7) | 0.0228 (8) | 0.0000 (7) |

*Geometric parameters (Å, º) for* ***8c***

| N1—C2 | 1.3635 (18) | C10—H10A | 0.9800 |
| --- | --- | --- | --- |
| N1—C8A | 1.3816 (19) | C10—H10B | 0.9800 |
| N1—H1 | 0.870 (15) | C10—H10C | 0.9800 |
| C2—O2 | 1.2628 (17) | N11—N12 | 1.3753 (16) |
| C2—C3 | 1.4255 (19) | N11—H11 | 0.876 (14) |
| C3—C4 | 1.3627 (19) | N12—C13 | 1.2659 (19) |
| C3—H3 | 0.9500 | C13—C14 | 1.4966 (19) |
| C4—N11 | 1.3824 (18) | C13—H13 | 0.9500 |
| C4—C4A | 1.4558 (19) | C14—C15 | 1.508 (2) |
| C4A—C5 | 1.4039 (19) | C14—H14A | 0.9900 |
| C4A—C8A | 1.411 (2) | C14—H14B | 0.9900 |
| C5—C6 | 1.381 (2) | C15—O15 | 1.2050 (19) |
| C5—H5 | 0.9500 | C15—O16 | 1.3371 (18) |
| C6—O9 | 1.3711 (18) | O16—C17 | 1.4486 (19) |
| C6—C7 | 1.406 (2) | C17—C18 | 1.505 (2) |
| C7—C8 | 1.383 (2) | C17—H17A | 0.9900 |
| C7—H7 | 0.9500 | C17—H17B | 0.9900 |
| C8—C8A | 1.4011 (19) | C18—H18A | 0.9800 |
| C8—H8 | 0.9500 | C18—H18B | 0.9800 |
| O9—C10 | 1.420 (2) | C18—H18C | 0.9800 |
|  |  |  |  |
| C2—N1—C8A | 123.69 (12) | H10A—C10—H10B | 109.5 |
| C2—N1—H1 | 117.2 (12) | O9—C10—H10C | 109.5 |
| C8A—N1—H1 | 118.7 (12) | H10A—C10—H10C | 109.5 |
| O2—C2—N1 | 119.79 (12) | H10B—C10—H10C | 109.5 |
| O2—C2—C3 | 122.85 (13) | N12—N11—C4 | 117.20 (11) |
| N1—C2—C3 | 117.31 (12) | N12—N11—H11 | 116.1 (12) |
| C4—C3—C2 | 121.67 (12) | C4—N11—H11 | 121.6 (12) |
| C4—C3—H3 | 119.2 | C13—N12—N11 | 115.86 (12) |
| C2—C3—H3 | 119.2 | N12—C13—C14 | 118.54 (13) |
| C3—C4—N11 | 122.54 (12) | N12—C13—H13 | 120.7 |
| C3—C4—C4A | 120.01 (12) | C14—C13—H13 | 120.7 |
| N11—C4—C4A | 117.45 (12) | C13—C14—C15 | 111.90 (12) |
| C5—C4A—C8A | 119.11 (13) | C13—C14—H14A | 109.2 |
| C5—C4A—C4 | 123.65 (12) | C15—C14—H14A | 109.2 |
| C8A—C4A—C4 | 117.24 (12) | C13—C14—H14B | 109.2 |
| C6—C5—C4A | 120.80 (13) | C15—C14—H14B | 109.2 |
| C6—C5—H5 | 119.6 | H14A—C14—H14B | 107.9 |
| C4A—C5—H5 | 119.6 | O15—C15—O16 | 123.97 (14) |
| O9—C6—C5 | 115.52 (13) | O15—C15—C14 | 125.39 (14) |
| O9—C6—C7 | 124.53 (13) | O16—C15—C14 | 110.63 (12) |
| C5—C6—C7 | 119.94 (13) | C15—O16—C17 | 115.14 (12) |
| C8—C7—C6 | 119.93 (13) | O16—C17—C18 | 107.12 (13) |
| C8—C7—H7 | 120.0 | O16—C17—H17A | 110.3 |
| C6—C7—H7 | 120.0 | C18—C17—H17A | 110.3 |
| C7—C8—C8A | 120.61 (13) | O16—C17—H17B | 110.3 |
| C7—C8—H8 | 119.7 | C18—C17—H17B | 110.3 |
| C8A—C8—H8 | 119.7 | H17A—C17—H17B | 108.5 |
| N1—C8A—C8 | 120.55 (13) | C17—C18—H18A | 109.5 |
| N1—C8A—C4A | 119.86 (12) | C17—C18—H18B | 109.5 |
| C8—C8A—C4A | 119.59 (13) | H18A—C18—H18B | 109.5 |
| C6—O9—C10 | 118.32 (13) | C17—C18—H18C | 109.5 |
| O9—C10—H10A | 109.5 | H18A—C18—H18C | 109.5 |
| O9—C10—H10B | 109.5 | H18B—C18—H18C | 109.5 |
|  |  |  |  |
| C8A—N1—C2—O2 | -178.97 (13) | C7—C8—C8A—N1 | 177.93 (13) |
| C8A—N1—C2—C3 | -1.3 (2) | C7—C8—C8A—C4A | -1.5 (2) |
| O2—C2—C3—C4 | 175.87 (13) | C5—C4A—C8A—N1 | -177.52 (12) |
| N1—C2—C3—C4 | -1.7 (2) | C4—C4A—C8A—N1 | 2.47 (19) |
| C2—C3—C4—N11 | -175.24 (12) | C5—C4A—C8A—C8 | 1.91 (19) |
| C2—C3—C4—C4A | 5.0 (2) | C4—C4A—C8A—C8 | -178.11 (12) |
| C3—C4—C4A—C5 | 174.65 (13) | C5—C6—O9—C10 | 172.46 (15) |
| N11—C4—C4A—C5 | -5.10 (19) | C7—C6—O9—C10 | -8.4 (2) |
| C3—C4—C4A—C8A | -5.34 (19) | C3—C4—N11—N12 | -1.1 (2) |
| N11—C4—C4A—C8A | 174.91 (12) | C4A—C4—N11—N12 | 178.63 (11) |
| C8A—C4A—C5—C6 | -1.0 (2) | C4—N11—N12—C13 | 156.97 (13) |
| C4—C4A—C5—C6 | 178.98 (13) | N11—N12—C13—C14 | 179.67 (12) |
| C4A—C5—C6—O9 | 178.90 (12) | N12—C13—C14—C15 | 99.52 (16) |
| C4A—C5—C6—C7 | -0.3 (2) | C13—C14—C15—O15 | -5.3 (2) |
| O9—C6—C7—C8 | -178.38 (13) | C13—C14—C15—O16 | 175.93 (12) |
| C5—C6—C7—C8 | 0.7 (2) | O15—C15—O16—C17 | -2.9 (2) |
| C6—C7—C8—C8A | 0.2 (2) | C14—C15—O16—C17 | 175.92 (11) |
| C2—N1—C8A—C8 | -178.59 (12) | C15—O16—C17—C18 | -179.08 (12) |
| C2—N1—C8A—C4A | 0.8 (2) |  |  |

*Hydrogen-bond geometry (Å, º) for* ***8c***

| *D*—H···*A* | *D*—H | H···*A* | *D*···*A* | *D*—H···*A* |
| --- | --- | --- | --- | --- |
| N1—H1···O2i | 0.87 (2) | 2.01 (2) | 2.8219 (16) | 154 (2) |
| N11—H11···O2ii | 0.88 (1) | 2.08 (2) | 2.9003 (16) | 155 (2) |
| C13—H13···O2ii | 0.95 | 2.60 | 3.3397 (18) | 135 |

Symmetry codes: (i) -*x*+1, -*y*+2, -*z*+1; (ii) *x*, *y*-1, *z*.





**Figure SI3**. X-Ray crystallographic structure structure of compound **8d** ((*E*)-Ethyl 3-(2-(6-methyl-2-oxo-1,2-dihydroquinolin-4-yl)hydrazono)propanoate) (solvent omitted, displacement parameters are drawn at 30% probability level).

*Crystal data for* ***8d***

| C15H17N3O·1.5(H2O) | *F*(000) = 1336 |
| --- | --- |
| *Mr* = 314.34 | *D*x = 1.287 Mg m-3 |
| Monoclinic, *C*2/*c (no.15)* | Cu *K*α radiation,  = 1.54178 Å |
| *a* = 17.8791 (3) Å | Cell parameters from 9906 reflections |
| *b* = 11.1773 (2) Å |  = 4.6–72.2° |
| *c* = 16.4921 (2) Å |  = 0.80 mm-1 |
| β = 100.178 (1)° | *T* = 298 K |
| *V* = 3243.92 (9) Å3 | Blocks, yellow |
| *Z* = 8 | 0.35 × 0.25 × 0.20 mm |

*Data collection for* ***8d***

| Bruker D8 VENTURE diffractometer with PhotonII CPAD detector | 2799 reflections with *I* > 2σ(*I*) |
| --- | --- |
| Radiation source: INCOATEC microfocus sealed tube | *R*int = 0.056 |
| rotation in  and , 1°, shutterless scans | max = 72.2°, min = 4.7° |
| Absorption correction: multi-scan  *SADABS* (Sheldrick, 2014) | *h* = -2222 |
| *T*min = 0.620, *T*max = 0.864 | *k* = -1310 |
| 16724 measured reflections | *l* = -2020 |
| 3185 independent reflections |  |

*Refinement for* ***8d***

| Refinement on *F*2 | Primary atom site location: dual |
| --- | --- |
| Least-squares matrix: full | Secondary atom site location: difference Fourier map |
| *R*[*F*2 > 2σ(*F*2)] = 0.069 | Hydrogen site location: mixed |
| *wR*(*F*2) = 0.215 | H atoms treated by a mixture of independent and constrained refinement |
| *S* = 1.08 | *w* = 1/[2(*F*o2) + (0.1217*P*)2 + 1.7518*P*]  where *P* = (*F*o2 + 2*F*c2)/3 |
| 3185 reflections | (Δ/σ)max < 0.001 |
| 214 parameters | max = 0.42 e Å-3 |
| 169 restraints | min = -0.44 e Å-3 |

*Fractional atomic coordinates and isotropic or equivalent isotropic displacement parameters (Å2) for* ***8d***

|  | *x* | *y* | *z* | *U*iso*/*U*eq |
| --- | --- | --- | --- | --- |
| N1 | 0.39907 (9) | 0.53980 (18) | 0.98876 (10) | 0.0603 (5) |
| H1 | 0.4377 | 0.5206 | 1.0253 | 0.072* |
| C2 | 0.40911 (11) | 0.5501 (2) | 0.90956 (11) | 0.0578 (5) |
| O2 | 0.47386 (8) | 0.53034 (18) | 0.89207 (9) | 0.0748 (5) |
| C3 | 0.34491 (11) | 0.58426 (19) | 0.85016 (11) | 0.0562 (5) |
| H3 | 0.3499 | 0.5912 | 0.7952 | 0.067* |
| C4 | 0.27585 (10) | 0.60717 (17) | 0.87209 (11) | 0.0520 (4) |
| C4A | 0.26623 (10) | 0.59055 (17) | 0.95655 (11) | 0.0507 (4) |
| C5 | 0.19706 (11) | 0.60547 (19) | 0.98489 (12) | 0.0573 (5) |
| H5 | 0.1538 | 0.6259 | 0.9473 | 0.069* |
| C6 | 0.19127 (12) | 0.59078 (19) | 1.06636 (12) | 0.0600 (5) |
| C7 | 0.25704 (13) | 0.5616 (2) | 1.12244 (12) | 0.0636 (5) |
| H7 | 0.2544 | 0.5532 | 1.1780 | 0.076* |
| C8 | 0.32523 (12) | 0.5452 (2) | 1.09706 (12) | 0.0635 (5) |
| H8 | 0.3682 | 0.5254 | 1.1352 | 0.076* |
| C8A | 0.33048 (11) | 0.55828 (18) | 1.01433 (11) | 0.0536 (5) |
| N9 | 0.21484 (9) | 0.64702 (17) | 0.81562 (9) | 0.0606 (5) |
| H9 | 0.1708 | 0.6557 | 0.8291 | 0.073* |
| N10 | 0.22608 (9) | 0.67243 (16) | 0.73763 (9) | 0.0588 (5) |
| C11 | 0.17267 (12) | 0.7237 (2) | 0.68985 (12) | 0.0641 (5) |
| H11 | 0.1276 | 0.7432 | 0.7076 | 0.077* |
| C12 | 0.18352 (12) | 0.7522 (2) | 0.60416 (12) | 0.0664 (6) |
| H12A | 0.1931 | 0.8373 | 0.6004 | 0.080* |
| H12B | 0.2280 | 0.7100 | 0.5930 | 0.080* |
| C13 | 0.11698 (12) | 0.7195 (2) | 0.53971 (12) | 0.0638 (5) |
| O13 | 0.05431 (10) | 0.6968 (2) | 0.55196 (10) | 0.0918 (6) |
| O14 | 0.13573 (9) | 0.7183 (2) | 0.46597 (9) | 0.0806 (6) |
| C14 | 0.07580 (16) | 0.6859 (4) | 0.39725 (15) | 0.1023 (11) |
| H14A | 0.0521 | 0.6115 | 0.4092 | 0.123* |
| H14B | 0.0372 | 0.7478 | 0.3887 | 0.123* |
| C15 | 0.1094 (2) | 0.6727 (6) | 0.3245 (2) | 0.151 (2) |
| H15A | 0.0707 | 0.6514 | 0.2786 | 0.227* |
| H15B | 0.1325 | 0.7468 | 0.3130 | 0.227* |
| H15C | 0.1473 | 0.6110 | 0.3334 | 0.227* |
| C16 | 0.11626 (14) | 0.6055 (3) | 1.09493 (16) | 0.0785 (7) |
| H16A | 0.0773 | 0.6232 | 1.0485 | 0.118* |
| H16B | 0.1199 | 0.6699 | 1.1340 | 0.118* |
| H16C | 0.1038 | 0.5328 | 1.1205 | 0.118* |
| O1W | 0.5000 | 0.4249 (3) | 0.7500 | 0.1033 (9) |
| H1W | 0.494 (3) | 0.470 (3) | 0.788 (2) | 0.155* |
| O2W | 0.05024 (16) | 0.6937 (4) | 0.83107 (18) | 0.1697 (18) |
| H2W1 | 0.037 (5) | 0.710 (6) | 0.875 (3) | 0.255* |
| H2W2 | 0.037 (5) | 0.623 (3) | 0.821 (5) | 0.255* |

*Atomic displacement parameters (Å2) for* ***8d***

|  | *U*11 | *U*22 | *U*33 | *U*12 | *U*13 | *U*23 |
| --- | --- | --- | --- | --- | --- | --- |
| N1 | 0.0428 (8) | 0.0923 (13) | 0.0440 (8) | 0.0081 (7) | 0.0028 (6) | 0.0094 (7) |
| C2 | 0.0469 (10) | 0.0804 (13) | 0.0461 (9) | 0.0036 (8) | 0.0081 (7) | 0.0059 (8) |
| O2 | 0.0473 (8) | 0.1274 (14) | 0.0507 (8) | 0.0150 (8) | 0.0111 (6) | 0.0125 (8) |
| C3 | 0.0521 (10) | 0.0758 (12) | 0.0405 (9) | 0.0061 (8) | 0.0076 (7) | 0.0059 (8) |
| C4 | 0.0473 (9) | 0.0636 (10) | 0.0434 (9) | 0.0038 (7) | 0.0036 (7) | 0.0006 (7) |
| C4A | 0.0460 (9) | 0.0612 (10) | 0.0445 (9) | 0.0028 (7) | 0.0069 (7) | 0.0003 (7) |
| C5 | 0.0479 (10) | 0.0727 (12) | 0.0511 (10) | 0.0053 (8) | 0.0079 (8) | -0.0004 (8) |
| C6 | 0.0569 (11) | 0.0696 (12) | 0.0561 (10) | 0.0017 (9) | 0.0170 (8) | -0.0023 (8) |
| C7 | 0.0676 (12) | 0.0795 (13) | 0.0457 (9) | 0.0022 (10) | 0.0152 (9) | 0.0045 (9) |
| C8 | 0.0578 (11) | 0.0853 (14) | 0.0460 (10) | 0.0042 (10) | 0.0054 (8) | 0.0084 (9) |
| C8A | 0.0479 (9) | 0.0677 (11) | 0.0446 (9) | 0.0011 (8) | 0.0063 (7) | 0.0031 (7) |
| N9 | 0.0479 (8) | 0.0894 (12) | 0.0438 (8) | 0.0131 (8) | 0.0065 (6) | 0.0059 (7) |
| N10 | 0.0545 (9) | 0.0767 (11) | 0.0432 (8) | 0.0079 (7) | 0.0028 (6) | 0.0026 (7) |
| C11 | 0.0552 (11) | 0.0836 (14) | 0.0515 (10) | 0.0121 (9) | 0.0038 (8) | 0.0044 (9) |
| C12 | 0.0591 (12) | 0.0853 (14) | 0.0518 (11) | 0.0038 (10) | 0.0018 (9) | 0.0108 (9) |
| C13 | 0.0548 (11) | 0.0851 (14) | 0.0508 (10) | 0.0060 (9) | 0.0075 (8) | 0.0082 (9) |
| O13 | 0.0599 (10) | 0.1554 (19) | 0.0608 (9) | -0.0084 (10) | 0.0129 (7) | -0.0003 (10) |
| O14 | 0.0562 (9) | 0.1355 (16) | 0.0492 (8) | -0.0028 (9) | 0.0069 (6) | 0.0037 (8) |
| C14 | 0.0634 (14) | 0.182 (3) | 0.0581 (13) | -0.0099 (17) | 0.0017 (11) | -0.0074 (16) |
| C15 | 0.094 (2) | 0.295 (7) | 0.0629 (16) | -0.027 (3) | 0.0092 (15) | -0.017 (2) |
| C16 | 0.0683 (13) | 0.1017 (18) | 0.0717 (14) | 0.0068 (12) | 0.0297 (11) | -0.0022 (12) |
| O1W | 0.128 (2) | 0.116 (2) | 0.0700 (16) | 0.000 | 0.0287 (17) | 0.000 |
| O2W | 0.0788 (15) | 0.342 (6) | 0.0922 (16) | 0.049 (2) | 0.0261 (13) | 0.007 (2) |

*Geometric parameters (Å, º) for* ***8d***

| N1—C2 | 1.354 (2) | N10—C11 | 1.263 (3) |
| --- | --- | --- | --- |
| N1—C8A | 1.381 (3) | C11—C12 | 1.494 (3) |
| N1—H1 | 0.8600 | C11—H11 | 0.9300 |
| C2—O2 | 1.261 (2) | C12—C13 | 1.494 (3) |
| C2—C3 | 1.423 (3) | C12—H12A | 0.9700 |
| C3—C4 | 1.371 (3) | C12—H12B | 0.9700 |
| C3—H3 | 0.9300 | C13—O13 | 1.200 (3) |
| C4—N9 | 1.377 (2) | C13—O14 | 1.317 (3) |
| C4—C4A | 1.445 (2) | O14—C14 | 1.461 (3) |
| C4A—C8A | 1.404 (3) | C14—C15 | 1.441 (5) |
| C4A—C5 | 1.407 (3) | C14—H14A | 0.9700 |
| C5—C6 | 1.376 (3) | C14—H14B | 0.9700 |
| C5—H5 | 0.9300 | C15—H15A | 0.9600 |
| C6—C7 | 1.400 (3) | C15—H15B | 0.9600 |
| C6—C16 | 1.507 (3) | C15—H15C | 0.9600 |
| C7—C8 | 1.369 (3) | C16—H16A | 0.9600 |
| C7—H7 | 0.9300 | C16—H16B | 0.9600 |
| C8—C8A | 1.392 (3) | C16—H16C | 0.9600 |
| C8—H8 | 0.9300 | O1W—H1W | 0.826 (10) |
| N9—N10 | 1.366 (2) | O2W—H2W1 | 0.823 (10) |
| N9—H9 | 0.8600 | O2W—H2W2 | 0.836 (10) |
|  |  |  |  |
| C2—N1—C8A | 123.92 (15) | C11—N10—N9 | 117.45 (17) |
| C2—N1—H1 | 118.0 | N10—C11—C12 | 118.47 (19) |
| C8A—N1—H1 | 118.0 | N10—C11—H11 | 120.8 |
| O2—C2—N1 | 119.29 (17) | C12—C11—H11 | 120.8 |
| O2—C2—C3 | 123.43 (17) | C13—C12—C11 | 113.61 (19) |
| N1—C2—C3 | 117.28 (17) | C13—C12—H12A | 108.8 |
| C4—C3—C2 | 121.54 (17) | C11—C12—H12A | 108.8 |
| C4—C3—H3 | 119.2 | C13—C12—H12B | 108.8 |
| C2—C3—H3 | 119.2 | C11—C12—H12B | 108.8 |
| C3—C4—N9 | 121.65 (16) | H12A—C12—H12B | 107.7 |
| C3—C4—C4A | 119.77 (16) | O13—C13—O14 | 123.4 (2) |
| N9—C4—C4A | 118.58 (16) | O13—C13—C12 | 125.6 (2) |
| C8A—C4A—C5 | 117.82 (17) | O14—C13—C12 | 111.02 (18) |
| C8A—C4A—C4 | 117.69 (16) | C13—O14—C14 | 116.56 (18) |
| C5—C4A—C4 | 124.49 (16) | C15—C14—O14 | 108.4 (2) |
| C6—C5—C4A | 122.19 (18) | C15—C14—H14A | 110.0 |
| C6—C5—H5 | 118.9 | O14—C14—H14A | 110.0 |
| C4A—C5—H5 | 118.9 | C15—C14—H14B | 110.0 |
| C5—C6—C7 | 118.26 (18) | O14—C14—H14B | 110.0 |
| C5—C6—C16 | 121.1 (2) | H14A—C14—H14B | 108.4 |
| C7—C6—C16 | 120.59 (19) | C14—C15—H15A | 109.5 |
| C8—C7—C6 | 121.23 (18) | C14—C15—H15B | 109.5 |
| C8—C7—H7 | 119.4 | H15A—C15—H15B | 109.5 |
| C6—C7—H7 | 119.4 | C14—C15—H15C | 109.5 |
| C7—C8—C8A | 120.24 (18) | H15A—C15—H15C | 109.5 |
| C7—C8—H8 | 119.9 | H15B—C15—H15C | 109.5 |
| C8A—C8—H8 | 119.9 | C6—C16—H16A | 109.5 |
| N1—C8A—C8 | 120.07 (17) | C6—C16—H16B | 109.5 |
| N1—C8A—C4A | 119.70 (16) | H16A—C16—H16B | 109.5 |
| C8—C8A—C4A | 120.23 (18) | C6—C16—H16C | 109.5 |
| N10—N9—C4 | 118.25 (15) | H16A—C16—H16C | 109.5 |
| N10—N9—H9 | 120.9 | H16B—C16—H16C | 109.5 |
| C4—N9—H9 | 120.9 | H2W1—O2W—H2W2 | 105 (3) |
|  |  |  |  |
| C8A—N1—C2—O2 | -179.3 (2) | C2—N1—C8A—C4A | -0.4 (3) |
| C8A—N1—C2—C3 | 1.2 (3) | C7—C8—C8A—N1 | -178.9 (2) |
| O2—C2—C3—C4 | -178.7 (2) | C7—C8—C8A—C4A | 1.2 (3) |
| N1—C2—C3—C4 | 0.7 (3) | C5—C4A—C8A—N1 | 178.39 (19) |
| C2—C3—C4—N9 | 176.1 (2) | C4—C4A—C8A—N1 | -2.1 (3) |
| C2—C3—C4—C4A | -3.3 (3) | C5—C4A—C8A—C8 | -1.8 (3) |
| C3—C4—C4A—C8A | 3.9 (3) | C4—C4A—C8A—C8 | 177.69 (19) |
| N9—C4—C4A—C8A | -175.47 (18) | C3—C4—N9—N10 | -5.0 (3) |
| C3—C4—C4A—C5 | -176.61 (19) | C4A—C4—N9—N10 | 174.42 (17) |
| N9—C4—C4A—C5 | 4.0 (3) | C4—N9—N10—C11 | -170.8 (2) |
| C8A—C4A—C5—C6 | 0.7 (3) | N9—N10—C11—C12 | -179.88 (19) |
| C4—C4A—C5—C6 | -178.71 (19) | N10—C11—C12—C13 | 134.2 (2) |
| C4A—C5—C6—C7 | 0.9 (3) | C11—C12—C13—O13 | 15.5 (4) |
| C4A—C5—C6—C16 | -179.1 (2) | C11—C12—C13—O14 | -164.4 (2) |
| C5—C6—C7—C8 | -1.5 (3) | O13—C13—O14—C14 | -0.7 (4) |
| C16—C6—C7—C8 | 178.5 (2) | C12—C13—O14—C14 | 179.2 (3) |
| C6—C7—C8—C8A | 0.4 (4) | C13—O14—C14—C15 | -171.8 (4) |
| C2—N1—C8A—C8 | 179.7 (2) |  |  |

*Hydrogen-bond geometry (Å, º) for* **8d**

| *D*—H···*A* | *D*—H | H···*A* | *D*···*A* | *D*—H···*A* |
| --- | --- | --- | --- | --- |
| N1—H1···O2i | 0.86 | 1.98 | 2.839 (2) | 178 |
| N9—H9···O2*W* | 0.86 | 2.20 | 3.044 (3) | 165 |
| O1*W*—H1*W*···O2 | 0.83 (1) | 1.94 (2) | 2.736 (2) | 163 (5) |
| O2*W*—H2*W*1···O13ii | 0.82 (1) | 2.20 (5) | 2.914 (3) | 146 (7) |

Symmetry codes: (i) -*x*+1, -*y*+1, -*z*+2; (ii) -*x*, *y*, -*z*+3/2.

**Spectral data**

Spectral data of compound **8a**

IR spectra (**8a**)


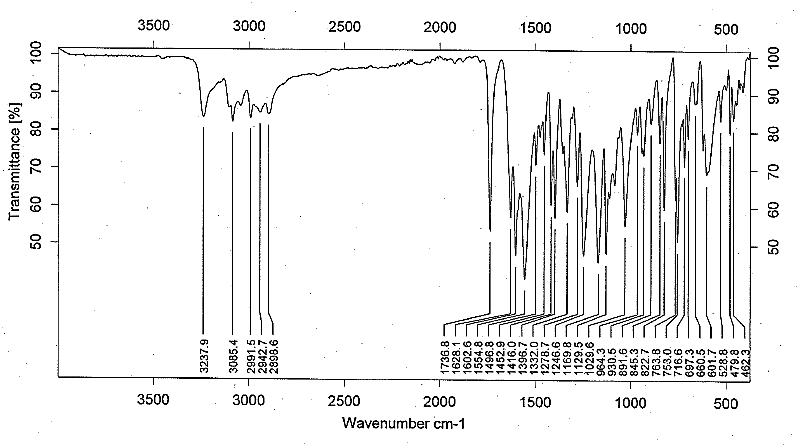


**Figure S14**

Mass spectrum (**8a**)


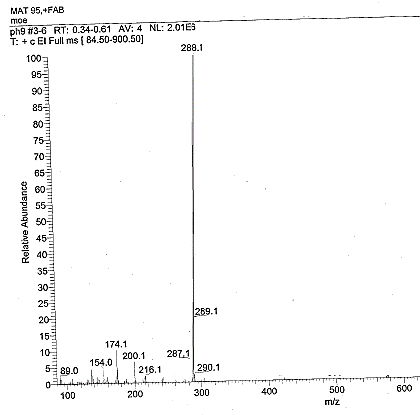


**Figure S15**

1H NMR (**8a**)


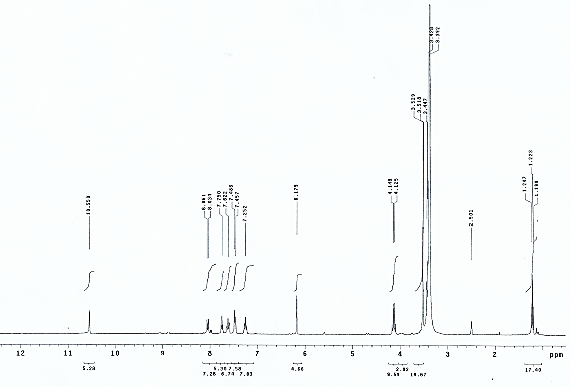


**Figure S16**

13C NMR (**8a**)


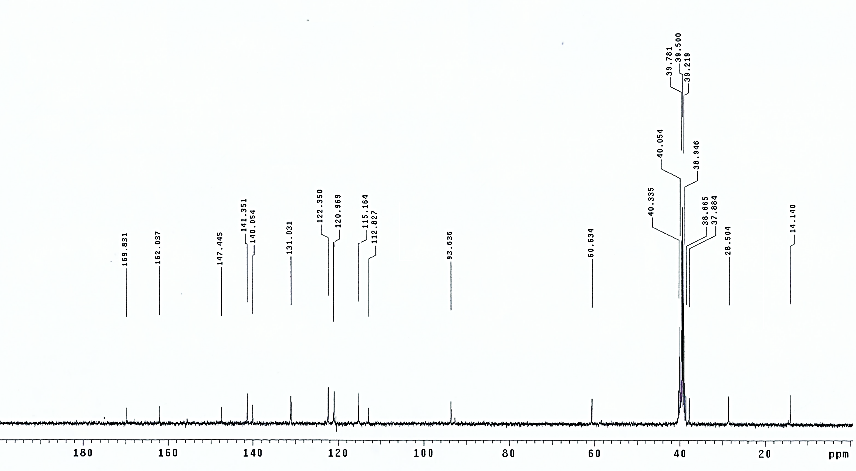


**Figure S17:** 13C NMR of compound **8a** (ethyl (*E*)-3-(2-(1-methyl-2-oxo-1,2-dihydroquinolin-4-yl)hydrazineylidene)propanoate)

Spectral data of compound **8b**

1H NMR (**8b**)


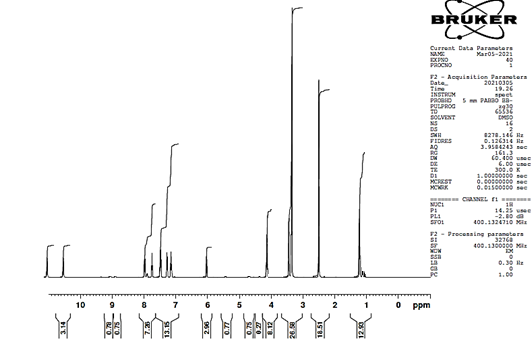


**Figure S18**

13C NMR (**8b**)


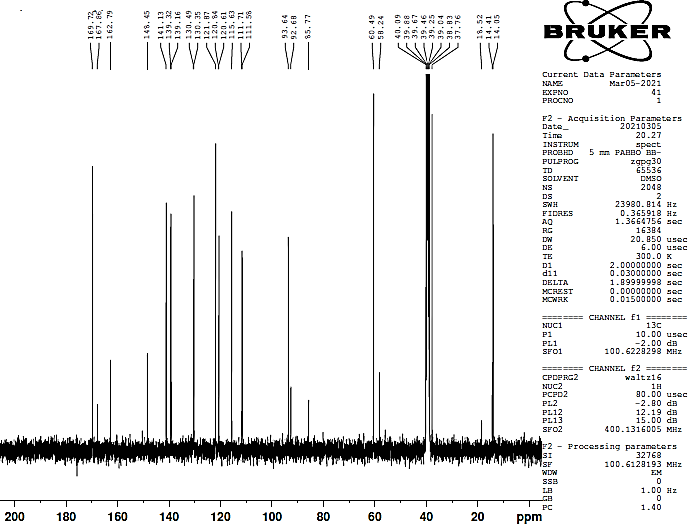


**Figure S19:** 13C NMR of compound **8b** (ethyl (*E*)-3-(2-(2-oxo-1,2-dihydroquinolin-4-yl)hydrazineylidene)propanoate)

Spectral data of compound **8c**

IR spectra (**8c**)


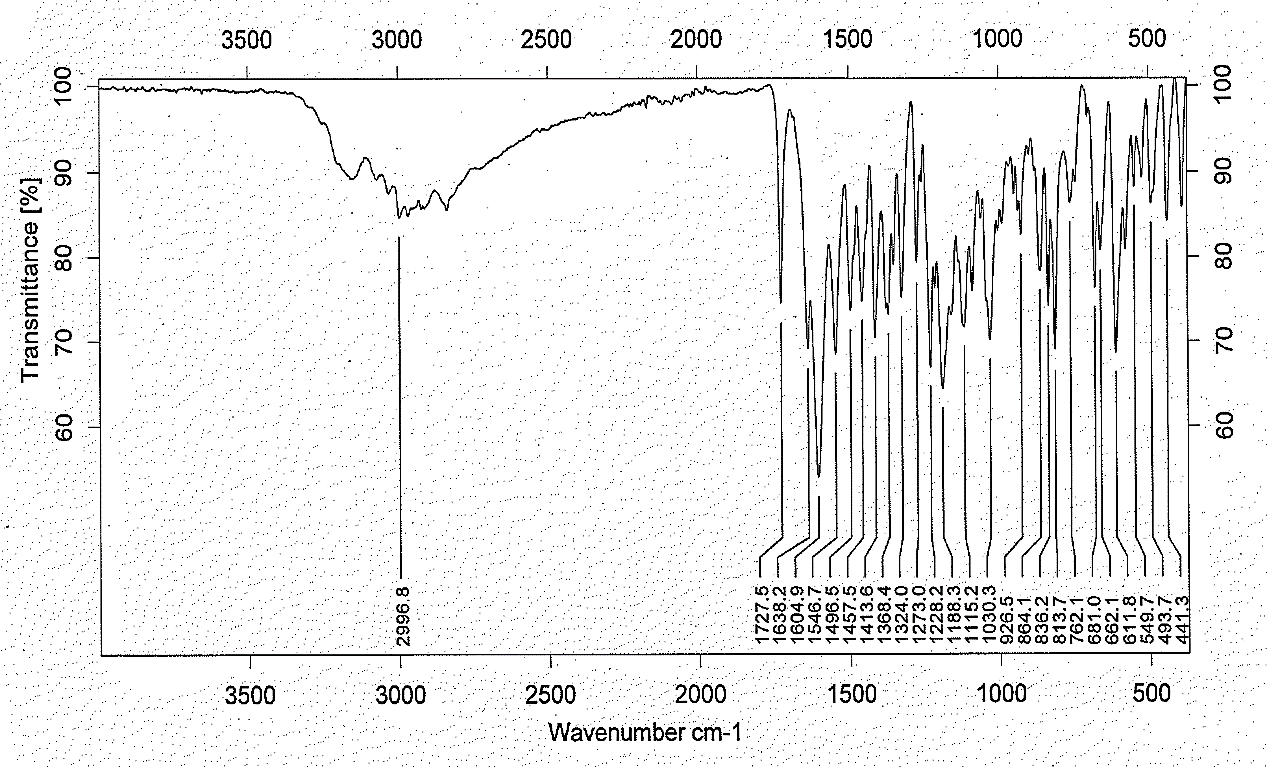


**Figure S20**

Mass spectrum (**8c**)


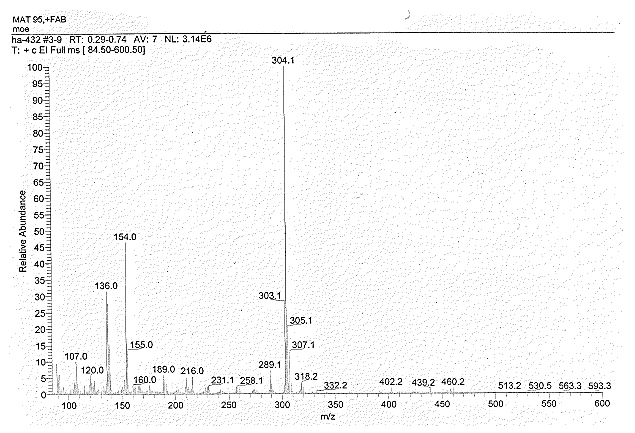


**Figure S21**

1H NMR (**8c**)


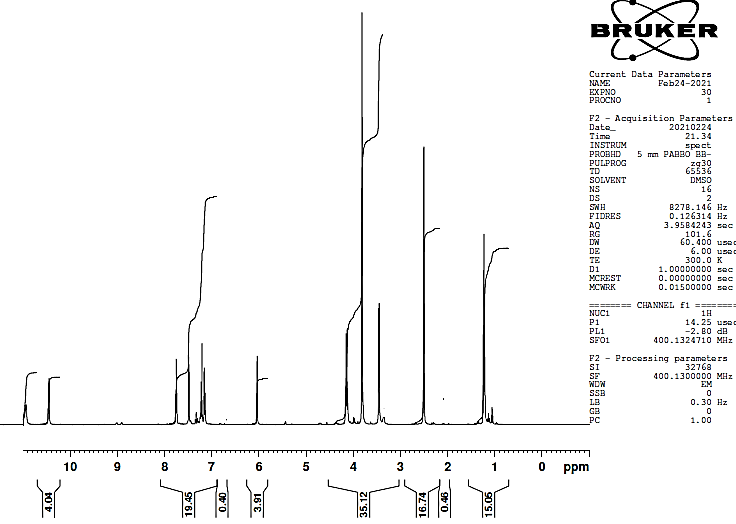


**Figure S22**

13C NMR (**8c**)


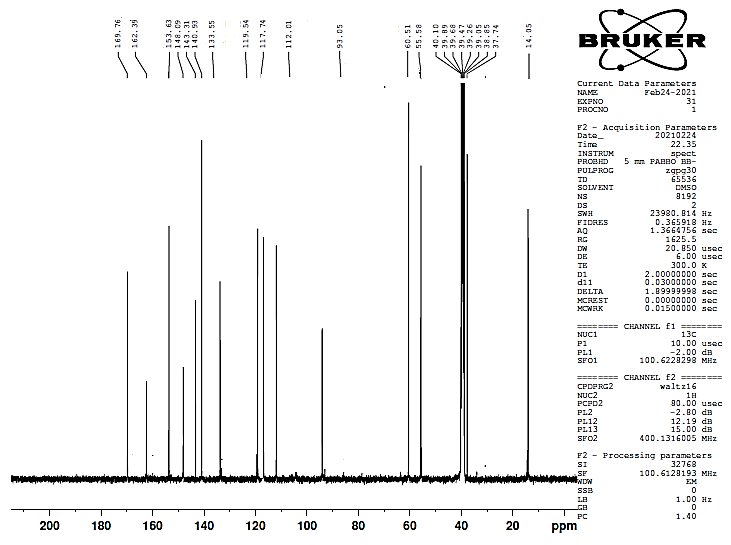


**Figure S23:** 13C NMR of compound **8c** (ethyl (*E*)-3-(2-(6-methoxy-2-oxo-1,2-dihydroquinolin-4-yl)hydrazineylidene)propanoate)

Spectral data of compound **8d**

1H NMR (**8d**)


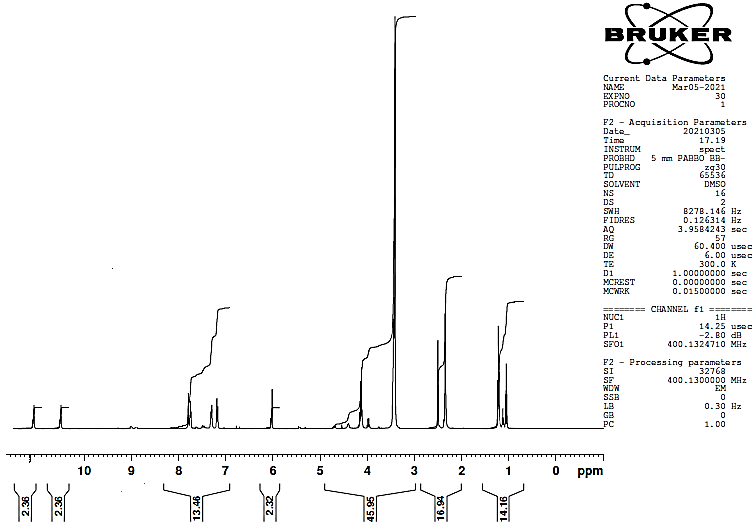


**Figure S24**

13C NMR (**8d**)


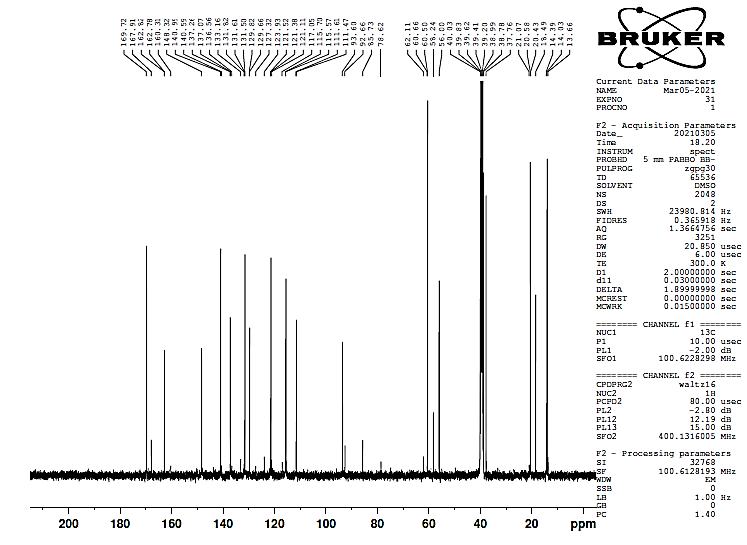


**Figure S25:** 13C NMR of compound **8d** (ethyl (*E*)-3-(2-(6-methyl-2-oxo-1,2-dihydroquinolin-4-yl)hydrazineylidene)propanoate)

Spectral data of compound **8e**

IR spectra (**8e**)


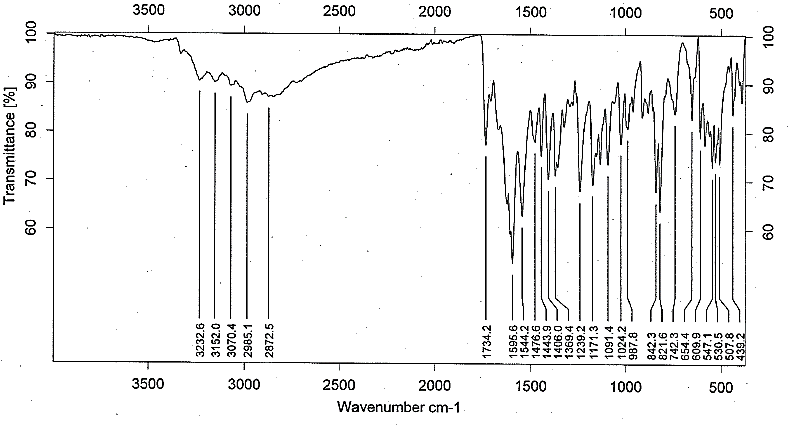


**Figure S26**

Mass spectrum (**8e**)


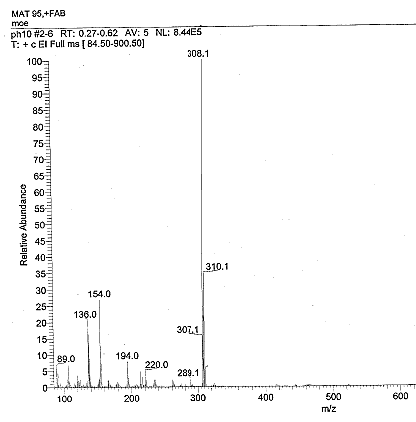


**Figure S27**

1H NMR (**8e**)


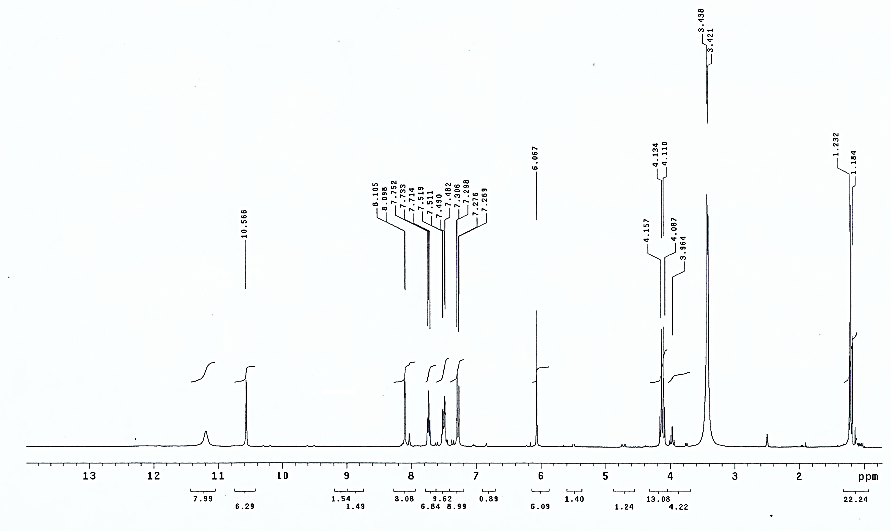


**Figure S28**

13C NMR (**8e**)


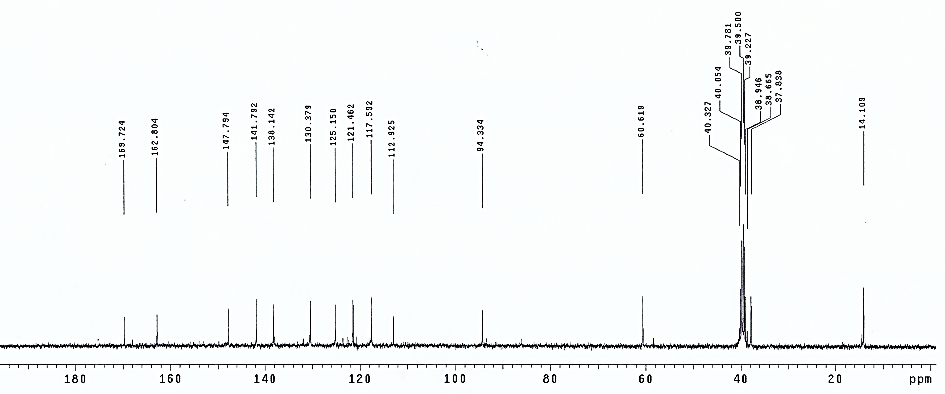


**Figure S29:** 13C NMR of compound **8e** (ethyl (*E*)-3-(2-(6-chloro-2-oxo-1,2-dihydroquinolin-4-yl)hydrazineylidene)propanoate)

Spectral data of compound **8f**

IR spectra (**8f**)


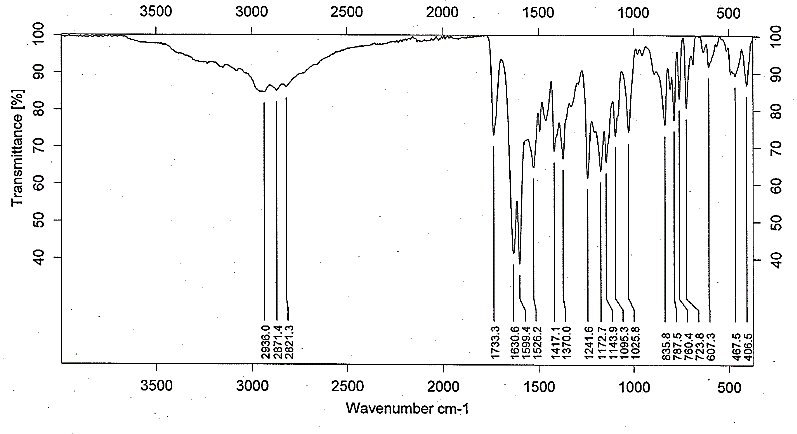


**Figure S30**

Mass spectrum (**8f**)


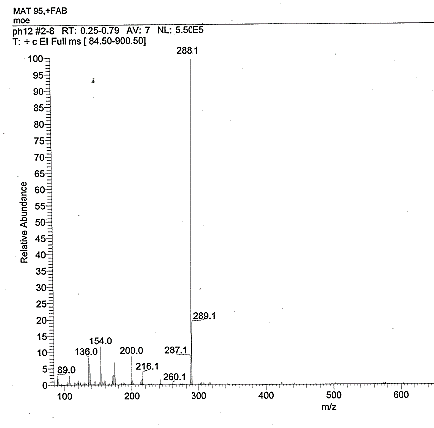


**Figure S31**

1H NMR (**8f**)


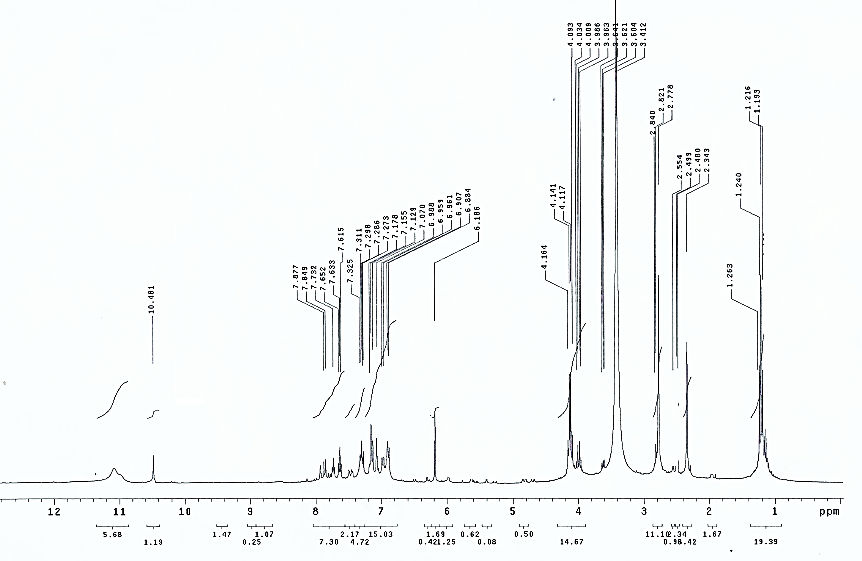


**Figure S32**

13C NMR (**8f**)


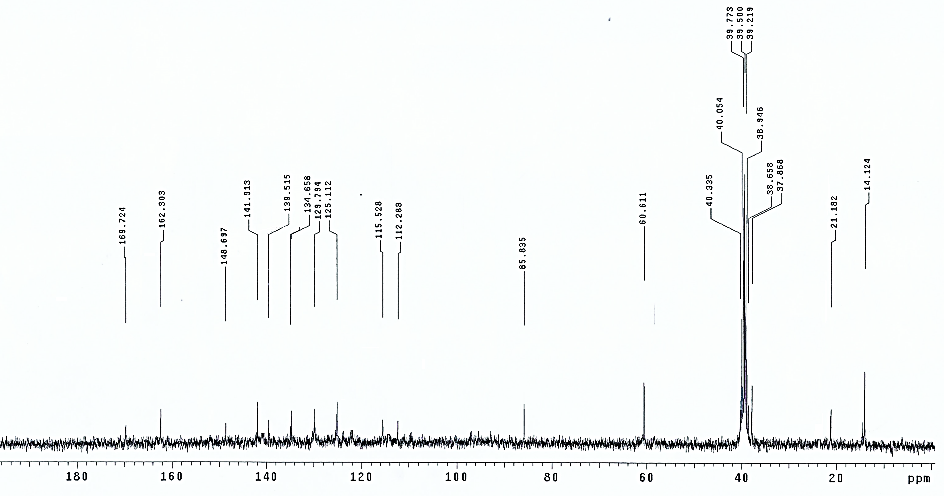


**Figure S33:** 13C NMR of compound **8f** (ethyl (*E)*-3-(2-(7-methyl-2-oxo-1,2-dihydroquinolin-4-yl)hydrazineylidene)propanoate)

Spectral data of compound **8g**

IR spectra (**8g**)


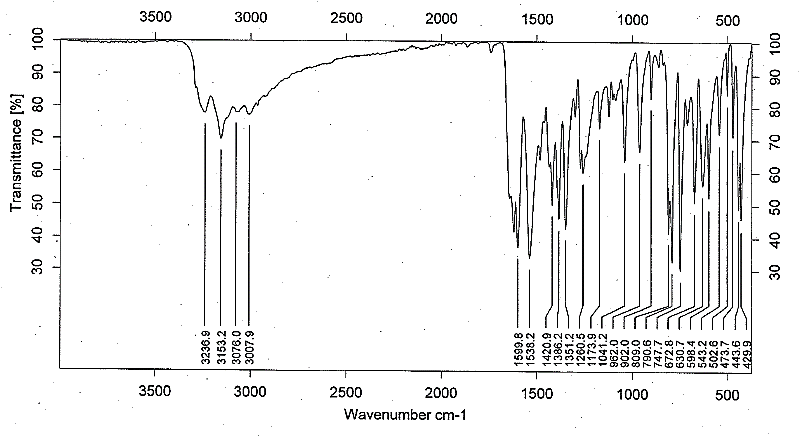


**Figure S34**

Mass spectrum (**8g**)


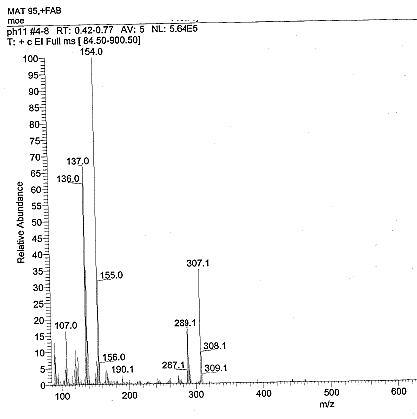


**Figure S35**

1H NMR (**8g**)


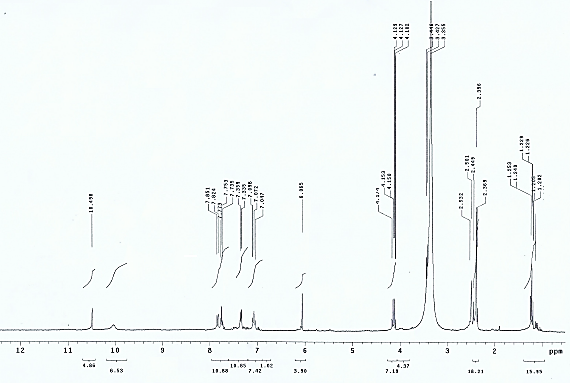


**Figure S36**

13C NMR (**8g**)


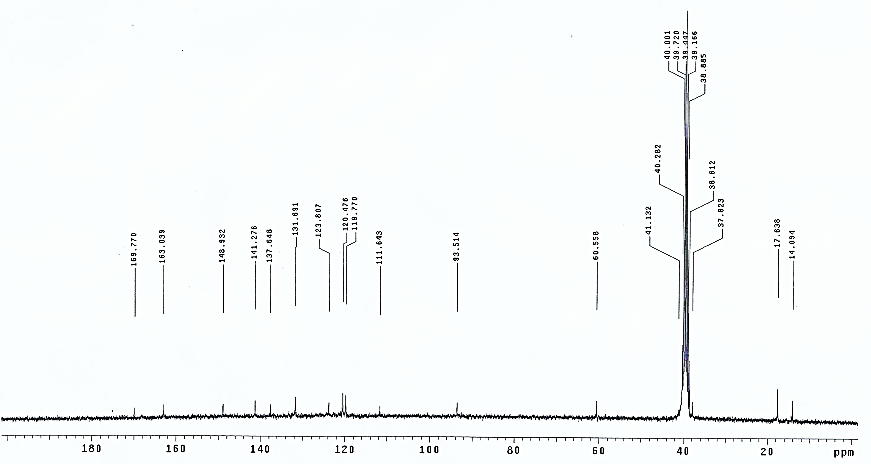


**Figure S37:** 13C NMR of compound **8g** (ethyl (*E*)-3-(2-(8-methyl-2-oxo-1,2-dihydroquinolin-4-yl)hydrazineylidene)propanoate).
